# Ontogenetic drivers of morphological evolution in monitor lizards and allies (Squamata: Paleoanguimorpha), a clade with extreme body size disparity

**DOI:** 10.1186/s12862-022-01970-6

**Published:** 2022-02-12

**Authors:** Carlos J. Pavón-Vázquez, Damien Esquerré, J. Scott Keogh

**Affiliations:** 1 Division of Ecology and Evolution, Research School of Biology, Australian National University, Canberra, ACT 2601 Australia; 2 Present Address: Department of Biological Sciences, New York City College of Technology, City University of New York, Brooklyn, NY 11201 USA

**Keywords:** Allometry, Morphometrics, Habitat use, Heterochrony, *Lanthanotus*, Ontogeny, *Shinisaurus*, *Varanus*

## Abstract

**Background:**

Heterochrony, change in the rate or timing of development, is thought to be one of the main drivers of morphological evolution, and allometry, trait scaling patterns imposed by size, is traditionally thought to represent an evolutionary constraint. However, recent studies suggest that the ontogenetic allometric trajectories describing how organisms change as they grow may be labile and adaptive. Here we investigated the role of postnatal ontogenetic development in the morphological diversification of Paleoanguimorpha, the monitor lizards and allies, a clade with extreme body size disparity. We obtained linear and geometric morphometric data for more than 1,600 specimens belonging to three families and 60 species, representing ~ 72% of extant paleoanguimorph diversity. We used these data to undertake one of the largest comparative studies of ontogenetic allometry to date.

**Results:**

Heterochrony is likely dictating morphological divergence at shallow evolutionary scales, while changes in the magnitude and direction of ontogenetic change are found mainly between major clades. Some patterns of ontogenetic variation and morphological disparity appear to reflect ontogenetic transitions in habitat use. Generally, juveniles are more similar to each other than adults, possibly because species that differ in ecology as adults are arboreal as juveniles. The magnitude of ontogenetic change follows evolutionary models where variation is constrained around an optimal value. Conversely, the direction of ontogenetic change may follow models with different adaptive optima per habitat use category or models where interspecific interactions influence its evolution. Finally, we found that the evolutionary rates of the ontogenetic allometric trajectories are phylogenetically variable.

**Conclusions:**

The attributes of ontogenetic allometric trajectories and their evolutionary rates are phylogenetically heterogeneous in Paleoanguimorpha. Both allometric constraints and ecological factors have shaped ontogeny in the group. Our study highlights the evolutionary lability and adaptability of postnatal ontogeny, and teases apart how different evolutionary shifts in ontogeny contribute to the generation of morphological diversity at different evolutionary scales.

**Supplementary Information:**

The online version contains supplementary material available at 10.1186/s12862-022-01970-6.

## Background

Evolutionary and ontogenetic changes in body size have huge consequences in many other traits [1, 2]. These size-related changes in phenotypic traits are referred to as allometry [2, 3]. Historically, allometry has been considered an evolutionary constraint as restrictions imposed by size are expected to limit the number of possible morphologies [4, 5]. There are three main approaches that have been employed to characterize allometric scaling: static allometry compares individuals belonging to the same species and developmental stage; ontogenetic allometry compares different developmental stages within a species, constituting the original definition of allometry [3]; and evolutionary allometry compares different species within the same developmental stage [6, 7]. Until recently, the evolution of ontogenetic allometry (i.e., the interspecific comparison of ontogenetic allometries) remained a comparatively understudied aspect of allometry. This approach has revealed that evolutionary shifts in ontogenetic allometries can occur at relatively shallow timescales, promoting morphological diversification. These shifts can be adaptive, reflecting the ecological characteristics of organisms [8–11].

**Fig. 1:**
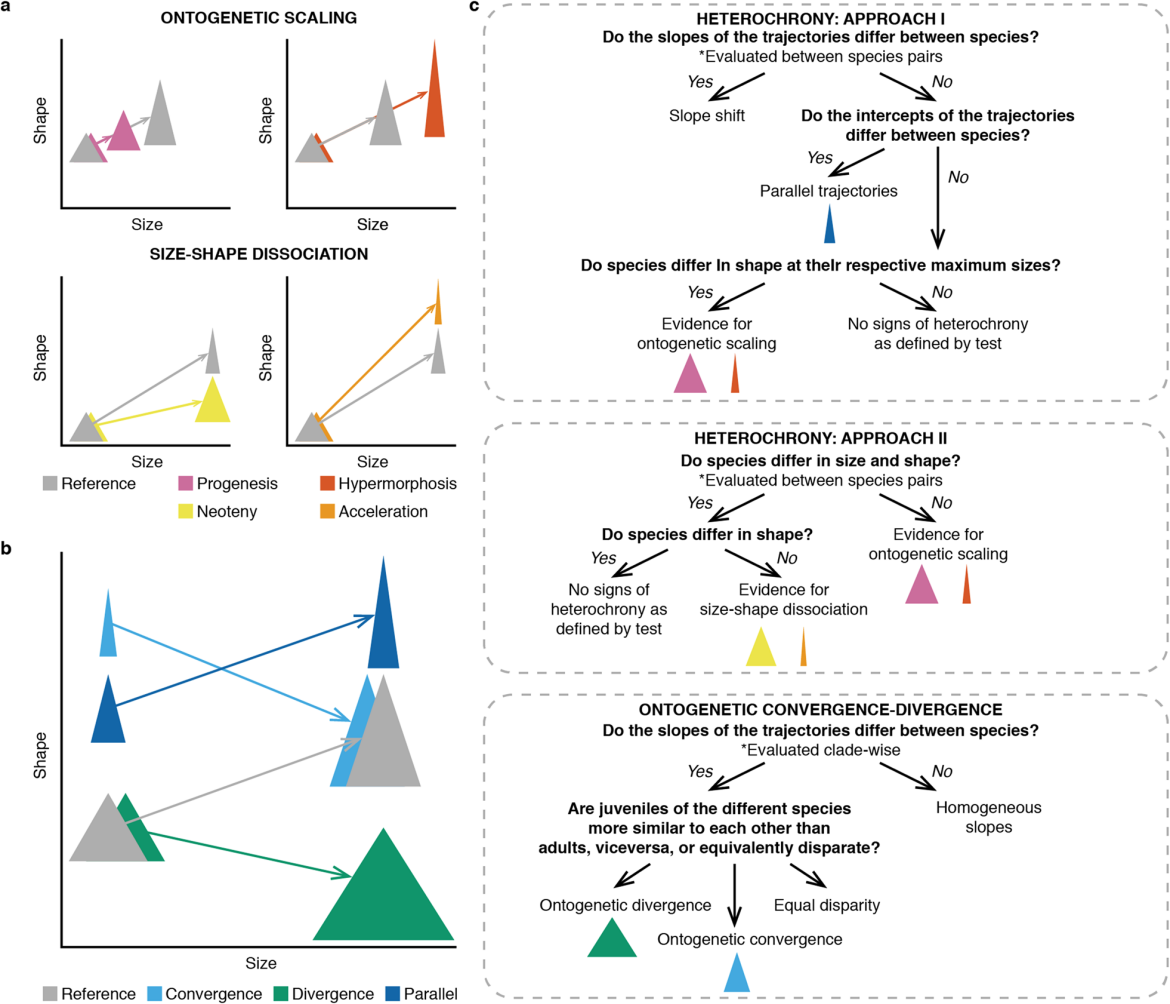
Evolutionary changes in ontogenetic allometric trajectories and approach used to identify them. **a** Heterochronic changes; trajectories are represented by arrows; triangles represent juvenile and adult shape, and are connected by arrows representing the trajectories. **b** Non-heterochronic changes. **c** Approach used to identify evolutionary shifts in the trajectories. Modified from Esquerré et al. [10] and Klingenberg [13]

Ontogenetic allometric trajectories can be conceptualized as vectors describing how shape changes with size through ontogeny (Fig. 1). The evolution of ontogenetic allometric trajectories can proceed in several ways. Traits that scale proportionally to body size are said to display isometry, while traits that scale disproportionally show allometry [2, 3]. Evolutionary shifts in the size-trait intercept when the direction of shape change with size remains constant produce non-overlapping parallel trajectories [12]. Trajectories can also diverge through heterochrony, understood as an evolutionary change in the rate or timing of developmental processes [13] that results in either paedomorphosis or peramorphosis [14]. Paedomorphic taxa exhibit a “juvenile-like” morphology compared to the ancestral phenotype, either through the early onset of maturity (progenesis) or a deacceleration of development (neoteny) [15]. Peramorphic taxa exhibit a more “adult-like” morphology, either through the late onset of maturity (hypermorphosis) or an acceleration of development (acceleration) [15]. Finally, evolution of ontogenetic allometric trajectories can involve shifts in the direction of phenotypic change. This can result in ontogenetic convergence, when adults of different taxa are more similar to each other than juveniles, or in ontogenetic divergence, when it is juveniles that are more similar to each other [8].

Paleoanguimorpha is a lizard clade with a distribution encompassing Africa, southern Asia, many Pacific islands, and Australia [16, 17]. The group is comprised of the families Shinisauridae, Lanthanotidae, and Varanidae. Shinisauridae and Lanthanotidae are represented by a single living species each: the Chinese crocodile lizard *Shinisaurus crocodilurus* from southeastern China and northern Vietnam [18], and the Borneo earless monitor *Lanthanotus borneensis* from Borneo [19]. Both of these taxa are poorly known inhabitants of riparian habitats. *Shinisaurus* regularly climbs overhanging vegetation [20], while *Lanthanotus* shelters in burrows or under leaf litter [19, 21]. In contrast, living monitor lizards (Varanidae) are classified into a single genus (*Varanus*), 11 subgenera, and around 80 species that show notable ecological and morphological diversity throughout their wide distribution [17, 22]. There is extreme size variation in Paleoanguimorpha. *Shinisaurus* and *Lanthanotus* average around 40 cm in total length [21, 23], the smallest monitor (*V*. *sparnus*) is only around 20 cm long [24], the largest living monitor (*V*. *komodoensis*) surpasses 3 m [25], and the colossal extinct *V*. *priscus* probably exceeded 5 m [26]. This makes *Varanus* the terrestrial vertebrate genus with the largest disparity in body size [27]. Additionally, many varanids undergo notable changes in size and ecology as they grow. For example, *V*. *komodoensis* hatchlings average only 42 cm in total length, are heavily arboreal, and feed on small prey. As they grow, they become strictly terrestrial and depend mainly on large ungulate prey [25, 28]. Other monitors experience similar ontogenetic transitions in diet and habitat use [29–33].

**Fig. 2:**
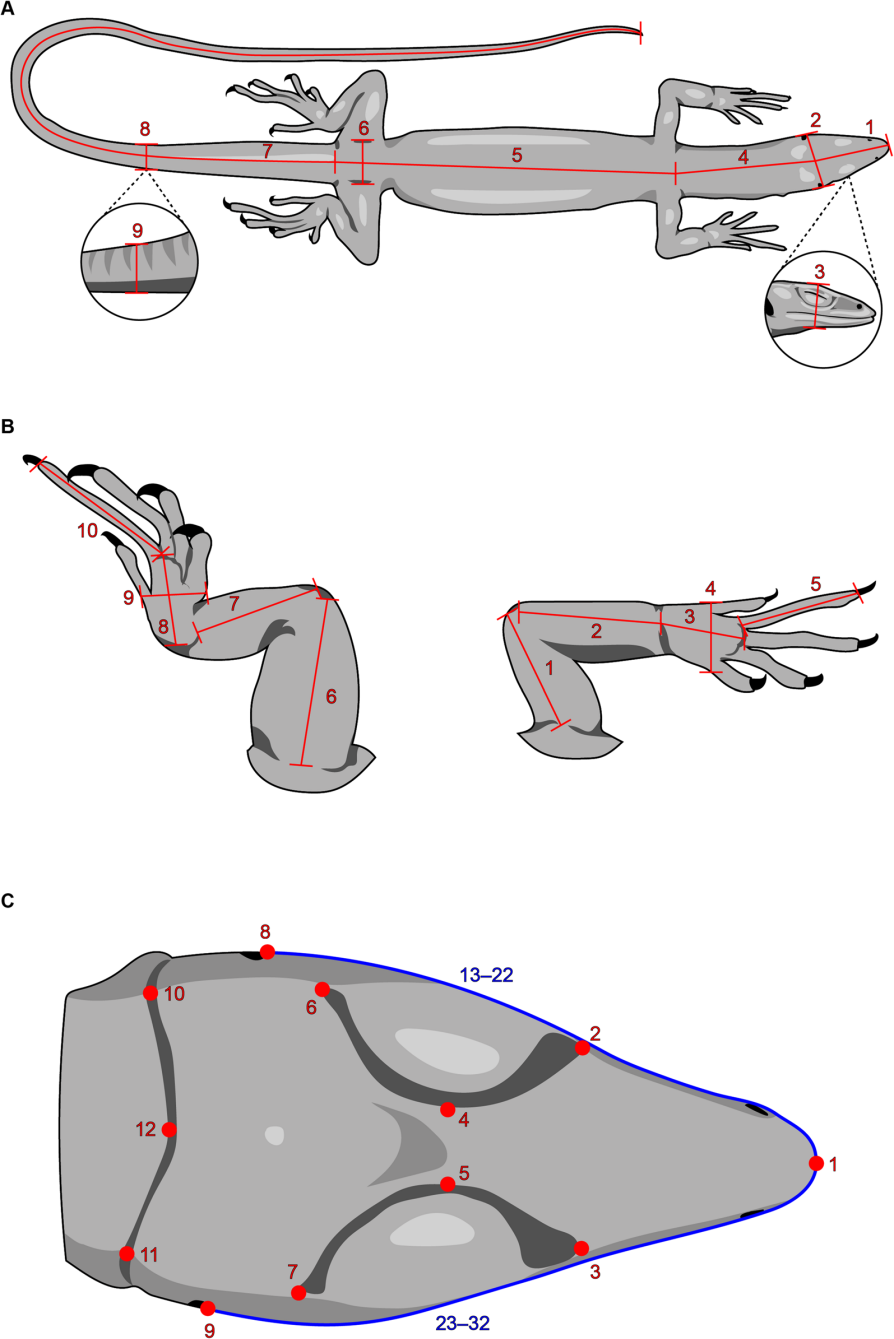
Morphometric data. **A** Linear measurements describing body shape: head length (1), head width (2), head depth (3), neck length (4), body length (5), hip width (6), tail length (7), tail width (8), and tail depth (9). **B** Linear measurements describing limb shape: upper arm length (1), lower arm length (2), hand length (3), hand width (4), finger IV length (5), upper leg length (6), lower leg length (7), foot length (8), foot width (9), and toe IV length (10). **C** Landmark configuration used to characterize head shape: tip of snout (1), anterior edges of supraocular semicircles (2–3), medial edges of supraocular semicircles (4–5), posterior edges of supraocular semicircles (6–7), anterior edges of tympanum (8–9), posterior edges of nuchal fold (10–11), and anterior edge of nuchal fold (12); the blue lines indicate sliding semi-landmarks (13–32)

The presence of ecological shifts and remarkable interspecific and ontogenetic size disparity make Paleoanguimorpha a suitable model to study how ontogenetic evolution drives morphological diversification. In this study, we measured over 1,600 specimens belonging to 60 living paleoanguimorph species to infer the macroevolutionary patterns of ontogenetic variation (Additional file 1: Table S1). We characterized ontogenetic changes in the shape of the body, limbs, and head through a combination of linear morphometrics (body and limbs) and two-dimensional geometric morphometrics (head) (Fig. 2; Additional file 1: Tables S2–S4; Additional file 2: Supporting methods). We evolutionarily contextualize our results based on a molecular phylogeny (Additional file 1: Table S5; Additional file 2: Fig. S1). Specifically, we ask: (1) what evolutionary ontogenetic changes are responsible for morphological differentiation at different timescales, (2) whether habitat use and associated ontogenetic shifts are reflected in evolutionary and ontogenetic allometries, and (3) what has been the tempo and mode of evolution of the ontogenetic allometric trajectories.

## Results

### Trajectory analyses

We characterized ontogenetic allometric trajectories through several approaches, performing independent analyses on each dataset. This allowed us to test the relevance of heterochronic and non-heterochronic developmental changes in the morphological differentiation of Paleoanguimorpha at different evolutionary scales. The body and limbs datasets consist of nine and ten measurements, respectively. Shape was described by log-shape ratios and the log-transformed geometric mean of all measurements was used as proxy for size. Head shape was characterized in dorsal view through 12 landmarks and 20 semi-landmarks. Analyses were based on the Procrustes-aligned coordinates and log-transformed centroid size was used as proxy for size.

**Fig. 3:**
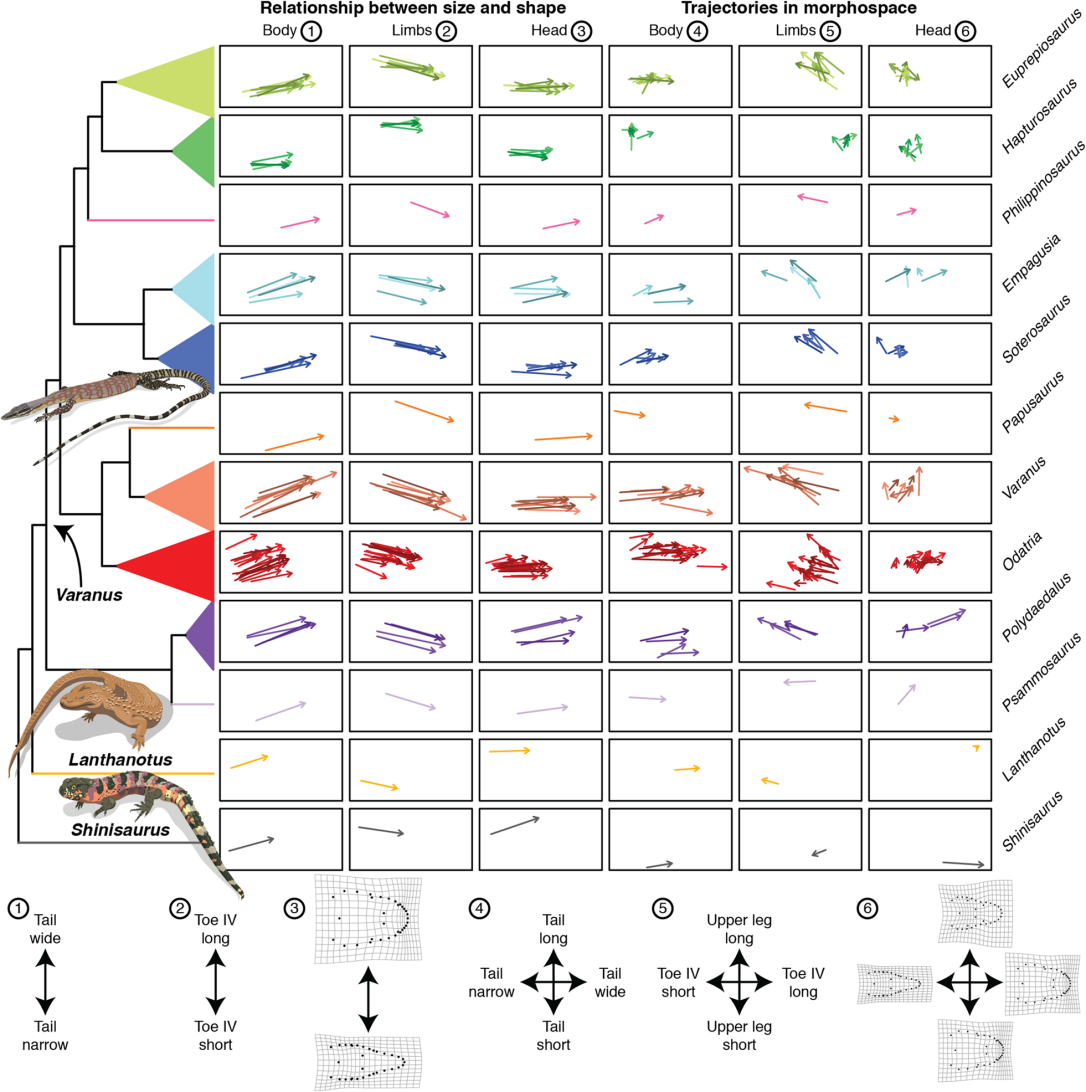
Ontogenetic allometric trajectories of Paleoanguimorpha. Each arrow represents the trajectory of a species as it grows. In the first three columns, the horizontal axis represents size (log-transformed geometric mean of linear measurements for body and limbs; log-transformed centroid size for head) and the vertical axes the first principal component (PC) of the predicted shape. In the last three columns, trajectories are plotted in morphospace: the horizontal and vertical axes represent the first and second PCs of the variables describing predicted shape, respectively. The tree (with arbitrary branch lengths) shows the relationships between the genera and subgenera within *Varanus*. The bottom diagrams are numbered in the same order as the columns and indicate the phenotypes at the extremes of each axis. The trait contributing the most to each PC is shown for the linear measurements and deformation grids of each extreme phenotype with respect to the average phenotype are shown for head shape

We fitted a linear model with shape as response variable and size as predictor to test whether each species displays isometric growth. Our results revealed that most species display allometric scaling, but numerous taxa are isometric for head shape (21 species; 8 for body shape and 6 for limb shape) (Additional file 1: Table S6). We performed a homogeneity of slopes test (HOST) to evaluate whether the slopes of the ontogenetic allometric trajectories are homogeneous in Paleoanguimorpha. The null hypothesis of homogeneous slopes was rejected across datasets (Additional file 1: Table S7). Instead, shape was strongly influenced by size, species, and their interaction (Additional file 1: Table S8). Thus, downstream analyses were based on trajectories resulting from regressions of shape on size performed separately for each species (i.e., each species has a unique allometry). The direction of ontogenetic change is somewhat conserved across species and mainly involves the relative widening of the tail, either lengthening or shortening of the tail, shortening of the fourth toe, elongation of the upper leg, and shortening and widening of the snout (Fig. 3; Additional file 2: Figs. S2–S8). Interestingly, a high proportion of species (78.57%) that transition in ecology from arboreal to terrestrial have relatively longer tails as juveniles (Additional file 1: Table S9), a trait that is associated with arboreality in varanids [34]. The same ontogenetic morphometric shift is found in 47.83% of the species that do not show ontogenetic shifts in habitat use. To summarize this developmental variation we visualized the ontogenetic allometric trajectories in two ways (Fig. 3): (1) plotting predicted shape against size, and (2) plotting how predicted shape changes through ontogeny in morphospace. Members of the highly arboreal varanid subgenus *Hapturosaurus* show some of the most distinctive trajectories, particularly in body and limb shape (Fig. 3).

**Fig. 4:**
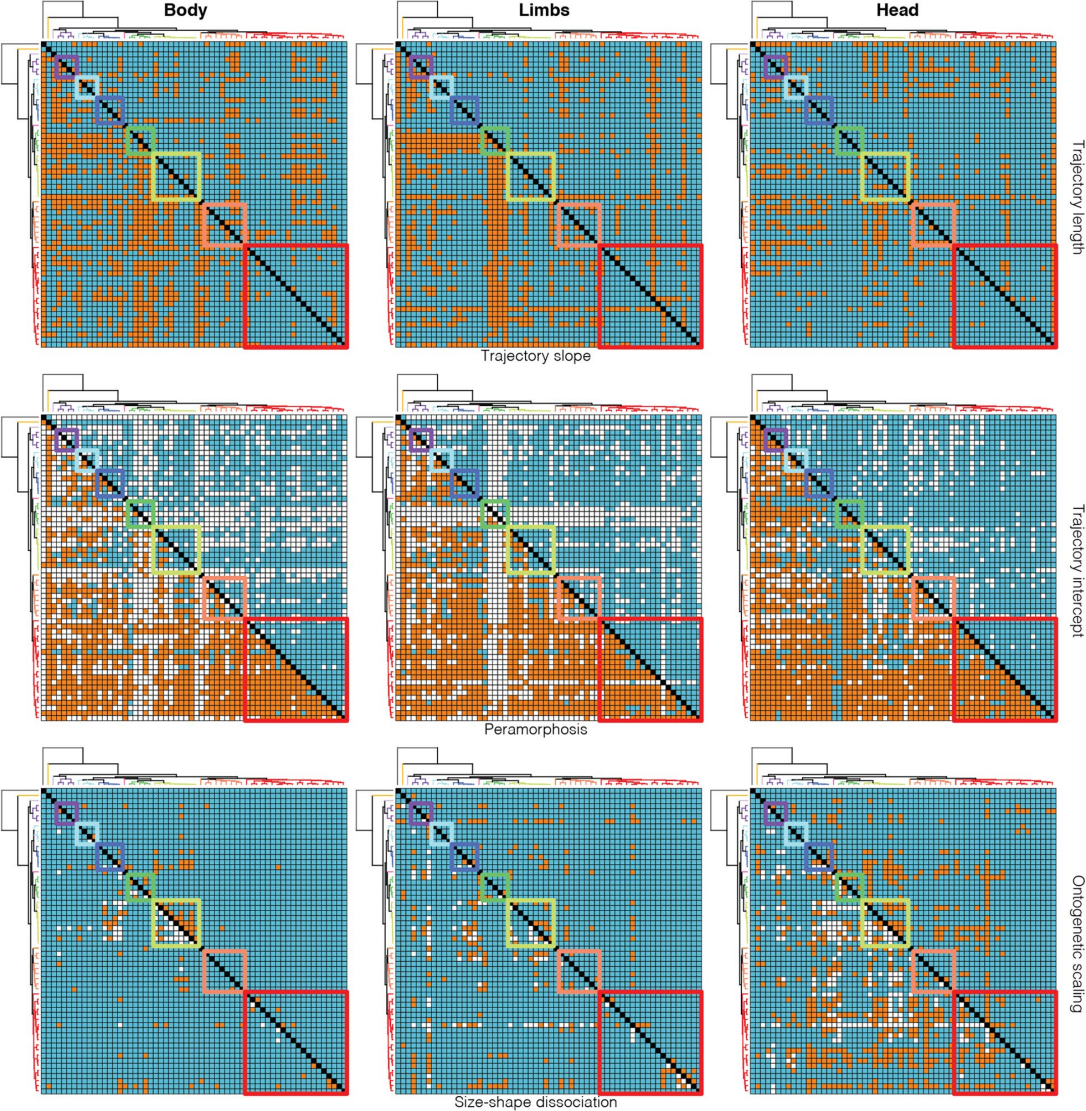
Phylogenetic patterns of variation in ontogenetic allometric trajectories and heterochrony in Paleoanguimorpha. Each grid is a square matrix where cells represent a pairwise comparison between species (diagonal in black). The phylogenetic tree depicting interspecific relationships is shown in the axes. Squares with colored borders indicate comparisons within clades (colors follow Fig. 3). White cells indicate comparisons that were not performed and blue cells represent negative results. Orange cells in the top row of grids indicate species pairs that differ significantly in trajectory length (upper triangle) or trajectory slope (lower triangle). Orange cells in the middle row indicate species pairs that have a common slope but differ significantly in intercept (upper triangle) or pairs that have a common slope and intercept but different adult shape, suggesting peramorphosis/paedomorphosis (lower triangle). In the upper triangle of the bottom row, orange cells indicate species pairs that overlap in shape and size-shape space, suggesting heterochrony by ontogenetic scaling. In the lower triangle, orange cells indicate species pairs that overlap in shape space but not in size-shape space, suggesting heterochrony by size-shape dissociation

We compared the length, slope, and intercept of ontogenetic allometric trajectories between species pairs to gain insight into phylogenetic patterns of ontogenetic variation and identify potential cases of heterochrony (Fig. 1). Significance was assessed either through residual randomization or permutation (Methods). We found numerous significant differences in the length and slope of trajectories between species pairs, both between and within clades (Fig. 4; Additional file 1: Tables S10–S17; Additional file 2: Figs. S9, S10). Head shape trajectories are more phylogenetically conserved than the trajectories of the body and limbs. Shifts in the length and slope of trajectories are more common between clades, but are also found within clades and between sister species. We then tested whether the species pairs sharing a common slope have intercepts that are either indistinguishable (potential heterochrony) or significantly different (parallel trajectories). This test revealed that none of the species pairs sharing a common slope have parallel trajectories, i.e., all species with indistinguishable slopes have indistinguishable intercepts (Additional file 1: Tables S18–S20).

Finally, we used hierarchical partitioning analyses to estimate the independent and joint effects of pairwise differences in trajectory attributes (angle, length, and intercept) on pairwise morphological disparity. Results (Additional file 1: Tables S21, S22) suggest that angle differences between the trajectories have the largest independent effect on adult morphological disparity in the body (explaining 77.26% of variance) and limbs (71.01%). Differences in the trajectory lengths explain most of the variance in adult head shape (52.27%).

### Heterochrony

The first approach used to detect heterochrony (hereafter “the peramorphosis test”) relies on the detection of significant differences in adult shape between species that share a common slope and intercept (Fig. 1). We found that most of these species pairs differ in adult morphology (Fig. 4; Additional file 1: Tables S23–S25). This indicates that paedomorphosis and peramorphosis are widespread in the group. Almost all comparisons with *Lanthanotus* were significant and the visualization of the trajectories shows that as varanids grow they move closer to the phenotype of *Lanthanotus* (Fig. 3), suggesting that Varanidae is paedomorphic with respect to its sister family Lanthanotidae. Heterochrony, as defined in this test, is more common than shifts in the slope of trajectories, especially within clades.

We used an alternative approach to detect heterochrony and identify cases of ontogenetic scaling and size-shape dissociation using Tfh1 and Tfh2 tests, respectively (Fig. 1). We first evaluated whether species pairs overlap in shape and size-shape space (i.e., species look the same and have the same size in a segment of their ontogenetic trajectory), with differentiation involving the extension or truncation of trajectories (ontogenetic scaling). In all datasets, we found evidence for ontogenetic scaling between multiple species pairs (Fig. 4; Additional file 1: Tables S26–S28). Amongst the species pairs that do not show ontogenetic scaling, we found multiple instances where taxa overlap exclusively in shape space (Fig. 4; Additional file 1: Tables S29–S31). These correspond to cases of heterochrony involving dissociation between size and shape. Of all datasets, body shape showed the least cases of either ontogenetic scaling or size-shape dissociation. These two kinds of heterochrony are found both between and within clades. Ontogenetic scaling was supported for 7, 9, and 8 pairs of sister species in the body, limbs, and head datasets, respectively. Heterochrony with size-shape dissociation was supported for three sister species pairs, all in the body dataset. Heterochrony between *Lanthanotus* and members of Varanidae more commonly involved size-shape dissociation, in agreement with the peramorphic phenotype but smaller size of *Lanthanotus*.

### Morphological variation in juveniles and adults

To identify clades displaying ontogenetic convergence/divergence (Fig. 1), we first used HOSTs to evaluate whether the species in Varanidae and each varanid subgenus with more than two species have a common allometric slope. The clade-specific HOSTs revealed that slopes are heterogeneous in most clades and datasets, except for limb shape in the varanid subgenera *Polydaedalus* and *Soterosaurus*, and head shape in the subgenus *Hapturosaurus* (Table 1; Additional file 1: Table S7). For the clades with heterogeneous slopes, including Paleoanguimorpha as a whole, we evaluated whether species show ontogenetic convergence or divergence using *D* as test statistic, which is obtained by subtracting a measure of morphological disparity among adults from the disparity exhibited by juveniles. A null distribution of *D* is obtained by randomizing the morphology of individuals with respect to their size. Across all datasets, we found evidence for ontogenetic divergence in Paleoanguimorpha and Varanidae (Table 1). Members of the subgenus *Empagusia* ontogenetically diverge in body shape, and those of *Odatria*, *Polydaedalus*, and *Soterosaurus* ontogenetically diverge in head shape. We found no significant instance of ontogenetic convergence.

**Table 1:** Test of ontogenetic convergence and divergence

Clade	Body			Limbs			Head		
	*p*-HOST	*D*	*p*-*D*	*p*-HOST	*D*	*p*-*D*	*p*-HOST	*D*	*p*-*D*
Paleoanguimorpha	< 0.001***	− 155.19	0.001***	0.002**	− 173.84	< 0.001***	< 0.001***	− 28.17	< 0.001***
Varanidae	< 0.001***	− 140.42	0.001***	0.003**	− 165.95	< 0.001***	< 0.001***	− 23.13	< 0.001***
*Empagusia*	0.001***	− 1.46	0.04*	0.01**	− 0.34	0.31	< 0.001***	0.04	0.36
*Euprepiosaurus*	0.01**	− 0.86	0.34	0.001***	− 0.86	0.31	< 0.001***	− 0.08	0.36
*Hapturosaurus*	0.03*	0.66	0.14	0.002**	0.4	0.08	0.10	–	–
*Odatria*	0.002**	3.57	0.32	0.01*	1.23	0.41	0.003**	− 2.52	< 0.001***
*Polydaedalus*	0.02*	0.55	0.23	0.11	–	–	0.03*	− 0.26	0.02*
*Soterosaurus*	0.02*	− 1.04	0.19	0.38	–	–	0.02*	− 0.16	0.02*
*Varanus*	< 0.001***	− 0.62	0.44	0.009**	0.46	0.44	< 0.001***	− 0.33	0.07

Analyses were performed on each clade for which the hypothesis of homogeneous slopes was rejected (*p*-HOST). For positive values of *D*, *p*-*D* is the proportion of permuted values of *D* that were greater than or equal to the empirical value. For negative values of *D*, *p*-*D* is the proportion of permuted values of *D* that were less than or equal to the empirical value. Asterisks indicate the significance of *p* values at different levels (*: *p* ≤ 0.05; **: *p* ≤ 0.01; ***: *p* ≤ 0.001)

We also evaluated the influence of phylogeny, size, and habitat use on juvenile and adult morphology. First, we calculated the phylogenetic signal of adult and juvenile morphology as a first approach to test whether phenotypic lability differs between them, which would suggest that each growth stage is subject to different evolutionary/ecological processes. The time-calibrated phylogeny that we used is primarily based on a genomic scale dataset (details in Methods and Additional file 2: Supporting methods). We calculated phylogenetic signal as a multivariate version of Blomberg’s *K* [35, 36] and calculated the difference in phylogenetic signal (Δ*K*) between adults (*K*_*a*_) and juveniles (*K*_*j*_). A null distribution of Δ*K* was obtained in the same way as for *D*. All morphological datasets displayed significant phylogenetic signal for both adults and juveniles (*p* < 0.001) (Additional file 1: Table S32). Adults displayed higher phylogenetic signal than juveniles (body: *K*_*a*_ = 0.29, juvenile *K*_*j*_ = 0.26; limbs: *K*_*a*_ = 0.26, *K*_*j*_ = 0.19; head: *K*_*a*_ = 0.50, *K*_*j*_ = 0.33). However, Δ*K* was only significant for head shape (body: Δ*K* = 0.03, *p* = 0.34; limbs: Δ*K* = 0.07, *p* = 0.56; head: Δ*K* = 0.16, *p* = 0.03). We used a phylogenetically informed MANOVA to test whether size, habitat use, or their interaction are influencing juvenile and/or adult morphology. We found significant deviation from Brownian motion in the relationship of body shape with habitat (*p* = 0.0002 in adults; *p* = 0.0003 in juveniles); limb shape with habitat (*p* = 0.0001 in adults and juveniles) and size (*p* = 0.001 in adults; *p* = 0.04 in juveniles); and head shape and size (*p* = 0.03 in adults; *p* = 0.02 in juveniles). Other results were not significant (Additional file 1: Table S33). Visualization of the phylomorphospace of adults and juveniles mirrors these results: phylogenetic clustering is apparent in all datasets, but clustering by habitat use is more evident in body shape and limb shape than in head shape (Additional file 2: Fig. S11).

### Evolution of trajectories

**Fig. 5:**
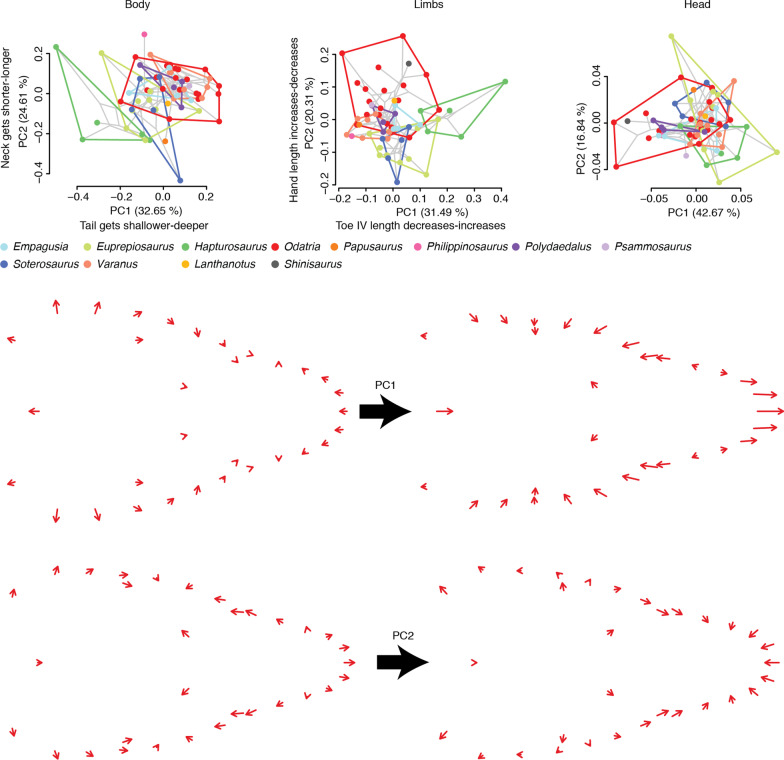
Phyloallomspace of Paleoanguimorpha. Axes correspond to the first two principal components (PCs) of the slopes of the ontogenetic allometric trajectories. The phylogenetic tree and inferred ancestral conditions (nodes) are shown in light gray. Convex hulls are shown for each clade. For the linear measurements, we show next to each axis the trait whose slope contributes majorly to each PC and how it changes ontogenetically at the lower and upper extremes (separated by dash, in that order). For head shape, we show below the average landmark configuration of juveniles (arrow origins) of the species at the extremes of each axis and how landmarks move as each species grows (arrow ends)

**Fig. 6:**
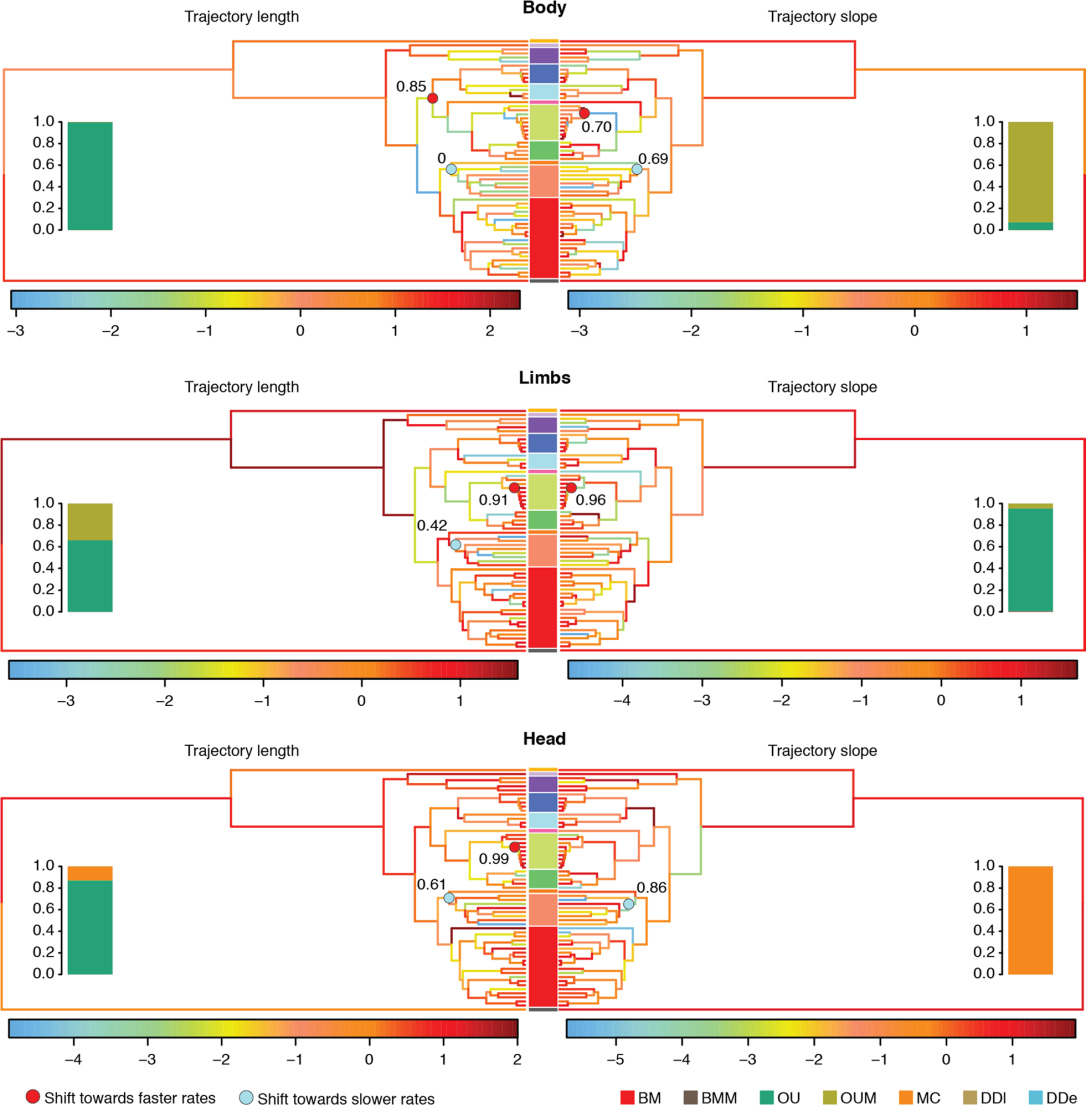
Evolution of ontogenetic allometric trajectories in Paleoanguimorpha. Rates of evolution of the length and slope of the trajectories are shown for each branch (natural logarithm of absolute rate). Colored bars between the mirrored trees indicate clades (colors follow Fig. 3). Circles indicate significant shifts and numbers indicate the proportion of trees with modified relationships, branch lengths, and taxon sampling in which these shifts were recovered. Bars indicate the support (AICcw) for different evolutionary models

To initially evaluate the lability of the length and slope of ontogenetic allometric trajectories we calculated their phylogenetic signal (*K*). We found no significant phylogenetic signal in the length of ontogenetic allometric trajectories (Additional file 1: Table S32). In contrast, we found significant phylogenetic signal in the slope of the trajectories (body: *K* = 0.08, *p* = 0.02; limbs: *K* = 0.09, *p* = 0.009; head: *K* = 0.08, *p* = 0.03) (Additional file 1: Table S32). We then performed the phylogenetic MANOVA procedure to assess the influence of adult size, habitat use, and their interaction on the trajectory attributes. We did not detect any significant influence of ecology or adult body size in trajectory lengths, and for the slopes we only found a significant relationship between the slopes of limb shape trajectories and adult body size (*p* = 0.03) (Additional file 1: Table S33). The strong influence of phylogeny and weaker influence of habitat use on the slopes can be visualized in the phyloallomspace, i.e., a two-dimensional plot of the first two PCs of the multivariate slopes of the ontogenetic allometric trajectories (Fig. 5; Additional file 2: Fig. S12). To infer the evolutionary mode of the ontogenetic allometric trajectories, we fitted evolutionary models to the trajectory lengths and first PC of the slopes. An Ornstein–Uhlenbeck (OU) model was supported for the length of ontogenetic allometric trajectories across all datasets (Fig. 6) (Additional file 1: Tables S34, S35). An OU model with multiple optima (OUM), one for each habitat use category, was preferred for the slope of the body trajectories. An OU model and a model where interspecific competition influences trait divergence (matching-competition model; MC) were strongly supported for the slope of the limb and head trajectories, respectively (Fig. 6; Additional file 1: Tables S35, S36).

As a final approach to characterize the evolution of postnatal ontogeny, we estimated branch-specific rates of evolution for the length and slope of trajectories. The evolutionary rates of the length and slope of the ontogenetic allometric trajectories are heterogeneous in Paleoanguimorpha (Fig. 6). Shifts towards faster rates were found mostly among the varanid subgenus *Euprepiosaurus*. Shifts towards slower rates were found among the clade containing the varanid subgenera *Papusaurus* and *Varanus*. The positive shifts were generally robust to phylogenetic uncertainty and sampling, in contrast with some of the negative shifts (Fig. 6).

## Discussion

### Heterochrony

Previous research has shown that morphological diversification may proceed through changes in the slope of ontogenetic allometric trajectories [8], their intercept [37], heterochrony [38], or a combination of these [10]. Heterochrony is thought to be one of the main drivers of morphological evolution [15, 39, 40]. Size-related phenotypic changes are pervasive and thus allometry acts as a strong integrating factor in body plans [41, 42]. This limits the amount of achievable morphological variation, with most evolutionary changes occurring along a path of least resistance that aligns with the slope of the allometric trajectories; i.e., the simplest way of attaining morphological modification is through a change in the timing or rate of development along an otherwise conserved allometric trajectory [39, 41, 42]. Thus, heterochronic evolutionary shifts are expected to occur more often at shallower timescales, while the slopes of the trajectories require more time to diverge [6, 40]. The results of the peramorphosis test align with this prediction: heterochronic changes are largely responsible for morphological evolution among closely related species. Significant interspecific differences in the magnitude and direction of ontogenetic change are more common between major clades, with the latter having the largest independent effect on body and limb shape variation. On the other hand, we did not detect any parallel trajectories showing a common slope but divergent intercept. The hierarchical partitioning analyses also suggest that intercept differences have a relatively small independent effect on morphological disparity. The approach based on the Tfh tests detected fewer instances of heterochrony, most of them in the head dataset. However, these analyses still suggest that heterochrony, and particularly ontogenetic scaling, has been an important driver of morphological differentiation. Such is the case of the *Euprepiosaurus* varanid subgenus and several pairs of sister species. In fact, ontogenetic scaling was supported for more sister species than heterochrony as defined by the peramorphosis test. Ours findings align with other results that support an important role for heterochrony at shallow evolutionary scales. In pythons, another reptile clade showing extreme body size disparity [10], heterochrony also seems to be responsible for morphological divergence at shallower scales, changes in the angle and length of slopes are more common at deeper scales, and significant intercept differences are uncommon.

Heterochrony has probably played a central role in some remarkable evolutionary transitions, such as the evolution of the avian and human skulls [15, 43]. Previous studies with limited sampling of taxa and traits have shown that heterochrony in Varanidae explains growth patterns [44], sexual dimorphism [45], and the huge size of the extinct *V*. *priscus* [46]. Our results show that heterochrony also has been an important driver of differentiation, mostly within but also between clades. Our study is limited by sampling, and biologically significant differences may not be statistically significant and vice versa. Thus, individual pairwise comparisons should be interpreted with caution, especially when they involve species with few sampled individuals. However, our sample sizes are relatively large for most species and the numerous pairwise comparisons increase power for the elucidation of phylogenetic patterns of ontogenetic variation. The type of heterochrony that appears to be more common in Paleoanguimorpha is one where morphological transformation follows changes in maximum adult size (as indicated by the peramorphosis test) (Fig. 4). Notably, heterochrony may be behind the origin of the varanid body plan. The length, slope, and intercept of the ontogenetic allometric trajectory of *Lanthanotus*, the sister of Varanidae, are similar to those of many varanid species (Fig. 4). However, adult shape differs markedly between them and, across datasets, the trajectories of most varanids move them closer to the phenotype of *Lanthanotus* as they grow. Thus, varanids seem to be paedomorphic with respect to *Lanthanotus*. However, *Lanthanotus* is smaller than most varanids. This is consistent with heterochrony by acceleration with size-shape dissociation. Accordingly, we found more instances of size-shape dissociation than of ontogenetic scaling between *Lanthanotus* and varanids.

The clade containing the largest varanids (*Varanus* + *Papusaurus*) is sister to that containing the smallest (*Odatria*), providing a unique opportunity to examine the association between size, shape, and ontogeny. Many pairwise differences in body shape between the two clades are mainly the result of shifts in the slope of the ontogenetic allometric trajectories (Fig. 4). Differences in the limbs and head are mainly driven by heterochrony, particularly through size-shape dissociation in the case of the head. In general, limb proportions are paedomorphic in the miniaturized *Odatria*. In contrast, head shape appears to be peramorphic in *Odatria*, explaining the support for size-shape dissociation. The smallest species included in our sampling, *V*. *brevicauda*, is peramorphic across datasets with respect to its most closely related species in this study, *V*. *eremius*, and also with respect to some members of the *Varanus* + *Papusaurus* clade. In fact, *V*. *brevicauda* has the most “adult-like” body shape of all sampled paleoanguimorphs (Fig. 3). The peramorphosis of *Lanthanotus*, *V*. *brevicauda*, and head shape in *Odatria* may seem surprising. However, instances of peramorphosis in miniaturized taxa and paedomorphosis in giants have been noted before, such as skull hyperossification in miniaturized frogs [47] and paedomorphosis in the skull of giant sauropodomorph dinosaurs [48]. On the other hand, the morphology of the largest living varanid, *V*. *komodoensis*, appears to be the result of different processes acting on different body parts. Body shape has been differentiated between this species and its sister, *V*. *varius*, by a change in the slope of the ontogenetic allometric trajectory, while the limbs and head of *V*. *komodoensis* are peramorphic with respect to *V*. *varius*. *Varanus komodoensis* has the most “adult-like” limb proportions among paleoanguimorphs. Thus, heterochrony resulting in peramorphosis is largely responsible for the shape of the smallest and largest paleoanguimorphs.

### The ecology and evolution of ontogeny

Our results suggest that ontogenetic shifts in habitat use may impact morphological evolution. Disparity in the evolutionary patterns between life stages has been thoroughly documented in animal taxa that undergo metamorphosis, where life stages are expected to be subjected to extremely different selective pressures and vary independently [49–51]. However, evolutionary consequences of ontogenetic ecological shifts also have been demonstrated in taxa with more gradual ontogenetic change [8, 10]. The results of the ontogenetic convergence/divergence test suggest that niches differ intraspecifically between juveniles and adults. We found several instances of ontogenetic divergence across all datasets, indicating that juveniles belonging to different species are more similar to each other than adults and accordingly there is lower phylogenetic signal in juveniles across datasets, significantly for head shape. This could reflect the observation that species differing in adult ecology are arboreal when they are juveniles [17]. This transition occurs in species that are terrestrial or amphibious as adults, while arboreal and escarpment-dwelling species remain as such throughout their lives and share similar adaptations for vertical and acrobatic movement, such as long tails and narrow bodies [34, 52]. Thus, many species with differing adult ecologies benefit from similar adaptations for climbing as juveniles.

An ecological interpretation of the differing patterns between ontogenetic stages is further supported by the ontogenetic tail reduction experienced by many species that shift from trunk-dwelling to terrestrial or amphibious. The ecological relevance of body shape is also supported by the phylogenetic MANOVA, which suggests that shape is correlated with habitat use independently of size in both adults and juveniles. Furthermore, most species exhibit negative ontogenetic allometry in the length of the digits, another trait that is correlated with climbing performance in lizards [53]. Moreover, the lengths of the upper and lower hindlimbs exhibit positive ontogenetic allometry, which may provide an adaptive advantage in terrestrial habitats [34, 54]. However, more research is needed to understand whether these allometric changes result in improved performance or simply maintain function as body size increases. The phylogenetic MANOVA indeed suggests that size has an equivalent effect on limb and head shape across the habitat use categories. Adults show more variation in body size than juveniles, and so the higher morphological disparity of adults could simply be a consequence of traits scaling allometrically to retain performance. Our results are therefore not conclusive on whether differences in phylogenetic patterns of morphological variation between juvenile and adult paleoanguimorphs are driven by habitat use.

Our analyses of the trajectory attributes under a comparative framework offer insight into the tempo and mode of evolution of ontogenetic development. The magnitude of ontogenetic shape change follows a OU model, consistent with stabilizing selection limiting allometric variation [40, 55]. Furthermore, trajectory lengths did not display significant phylogenetic signal or a strong correlation with maximum adult size and/or ecology deviating from BM. This perhaps indicates that the magnitude of ontogenetic shape change is so conserved across Paleoanguimorpha that it is very similar among distantly related taxa, as also suggested by the pairwise comparisons (Fig. 4). On the other hand, the direction of ontogenetic shape change displays strong phylogenetic signal, suggesting that it has evolved but not as fast as to overcome phylogenetic effects. Additionally, some variation appears to reflect ecological diversity, since an evolutionary model with multiple adaptive optima best fits the body slopes, and interspecific competition appears to have driven the evolution of the head slopes as suggested by the support for the MC model. Furthermore, the specialized arboreal varanids of the *Hapturosaurus* subgenus show trajectories that are very distinctive (Figs. 3, 4). In some *Hapturosaurus* species, ontogenetic change goes in a different direction and the magnitude of change in the group is comparatively small. This is probably because while other species climb less often as they grow, members of *Hapturosaurus* remain highly arboreal. The specialized arboreal python *Morelia viridis* also shows a trajectory that differs markedly from other pythons [10]. However, the phylogenetic MANOVA did not detect any significant correlation between any of the trajectory attributes and habitat use. The regressions that do not account for phylogeny detected a significant relationship of habitat with the limb trajectory lengths and the trajectory angles of all datasets. This means that habitat use covaries with these trajectory attributes, but it is challenging to untangle the effects of common descent and selection.

There is substantial variation in the evolutionary rates of the trajectory lengths and slopes (Fig. 6). Positive shifts were found mostly in the *Euprepiosaurus* varanid subgenus, a group that has rapidly diversified with moderate ecological differentiation [56]. Moderately and strongly supported shifts towards slower rates were detected in a clade that includes the largest varanids, such as *V*. *giganteus*, *V*. *komodoensis*, *V*. *salvadorii*, and *V*. *varius*, suggesting that large size may be imposing constraints on the evolution of ontogenetic allometric trajectories. Gould [1] suggested that decreases in the slope of allometric trajectories are necessary to develop large body sizes, in order to avoid non-viable or maladaptive phenotypes in adults. However, a meta-analysis of static allometries did not support this prediction [40]. More research on the relationship between size, the attributes of ontogenetic allometric trajectories, and their evolutionary rates is needed to test Gould’s [1] hypothesis.

## Conclusions

Here we show that different types of ontogenetic shifts are responsible for morphological diversification in Paleoanguimorpha, a lizard group exhibiting extreme body size disparity and ontogenetic ecological shifts. Our study further confirms that postnatal ontogenetic development should be considered as an evolutionary labile and potentially adaptive attribute of organisms. The insight gained into our research questions can be summarized as follows:

1. What evolutionary ontogenetic changes are responsible for morphological differentiation at different timescales? Heterochrony has allowed these lizards to morphologically diversify along a path of least-evolutionary resistance, playing a central role in phenotypic evolution. Heterochrony may also be partly responsible for the origin of the varanid body plan and seems to be the main driver of evolution at shallow evolutionary scales. The magnitude and direction of ontogenetic change are more evolutionarily conserved, mostly distinguishing major clades.
2. Are habitat use and associated ontogenetic shifts reflected in evolutionary and ontogenetic allometries? Ecological factors may explain some of the variation in the angles of the ontogenetic allometric trajectories, as exemplified by the unique slopes of a highly specialized arboreal clade. Our results also suggest that ontogenetic shifts in habitat use may have evolutionary consequences. Selection favouring traits that enhance climbing performance may explain the phenotypic similarity of juveniles belonging to distantly related clades. In adults, selection for climbing appears to be relaxed and interspecific differences accentuate.
3. What has been the tempo and mode of evolution of the ontogenetic allometric trajectories? The evolutionary rates of the ontogenetic allometric trajectories are highly variable, and we detected several rate accelerations and slowdowns. The trajectory lengths of all datasets and the slope of the limb trajectories follow an evolutionary model where variation is restricted around an optimum. The slopes of the body and head datasets follow models that are influenced by habitat use and interspecific competition, respectively.

## Methods

### Taxonomic sampling

We obtained morphometric data for all three genera in Paleoanguimorpha and most of the 11 subgenera within *Varanus*, except for the monotypic *Solomonsaurus*. Taxonomic uncertainty in Varanidae required us to make decisions on what we are treating as taxonomic units in this study (Additional file 2: Supporting methods). We aimed to characterize the ontogenetic series of each sampled species, ranging from hatchlings to large adults. We did not include species for which we could not measure small juveniles and large adults, or obtain a sample size ≥ 5. In total, we analyzed 60 species (Additional file 1: Table S1).

### Morphometrics

All analyses were performed in R 3.6.2 [57]. We used a 95% significance level and, unless noted, accounted for the false discovery rate (type I error) by adjusting probability values (*p*) when performing large numbers of pairwise comparisons through the Benjamini–Hochberg procedure [58]. We obtained nine and ten linear measurements describing body and limb morphology, respectively (Fig. 2; Additional file 1: Tables S2, S3; Additional file 2: Supporting methods). We corrected for body size while retaining allometric effects using log-shape ratios. For this, we calculated individual size as the geometric mean of all the measurements (both datasets combined), divided each trait by size, and log-transformed the resulting ratios [59]. For each dataset, we assessed sexual dimorphism through an analysis of variance and, since our sampling is male-biased, discarded females of those species showing a significant effect of sex on morphology (Additional file 1: Table S1). Our final sampling included 1,676 specimens for the body dataset (5–132 per species, *x̄* = 27.93) and 1,720 for the limbs dataset (5–132 per species, *x̄* = 28.67). We characterized head shape using two-dimensional geometric morphometrics. We photographed the head in dorsal view and recorded 12 landmarks and 20 semi-landmarks (Fig. 2; Additional file 1: Table S4; Additional file 2: Supporting methods) that were sled based on the minimization of bending energy [60] in the ‘geomorph 3.0.3’ R package [61]. To remove the effects of size, location, and orientation we performed a generalized Procrustes analysis (GPA) [62], taking bilateral asymmetry into account and using the symmetric component of shape in subsequent analyses [63, 64]. We evaluated sexual dimorphism in the aligned coordinates using the procedure described above and removed females of dimorphic species, resulting in a total sample size of 1,654 specimens (5–127 per species, *x̄* = 27.5) (Additional file 1: Table S1). We then redid the GPA on the unaligned coordinates of the retained individuals. Across datasets, only two species have a sample size of five, 88.33% of species are represented by ten specimens or more, and more than 63% of species are represented by 20 specimens or more (Additional file 1: Table S1). Additional details on the recording and processing of morphological data are found in Additional file 2: Supporting methods.

### Trajectory analyses

We evaluated whether each species displays isometry or allometric scaling by fitting a linear model with shape as response variable and size as predictor [10] in ‘geomorph 4.0.0’ [65]. Size was defined as the log-transformed geometric mean of all linear measurements for the linear datasets and as the log-transformed centroid size for the head shape dataset. We assessed significance through residual randomization with 10,000 permutations. A significant relationship between size and shape indicates allometric scaling, while independence between size and shape suggests isometry. We performed a homogeneity of slopes test (HOST) to evaluate whether the ontogenetic allometric slopes differ between species [8, 66]. To do this, we fitted two nested linear regressions with the “procD.lm” function of geomorph, assessing significance through 10,000 residual randomization iterations. The first was a multiple regression where shape was specified as response variable, while size and species were treated as independent predictors. The second indicates a model where the species trajectories are unique by adding the interaction between size and species as predictor. We then used the “anova” function of ‘RRPP 1.0.0’ [67, 68] to compare the models through their *F* statistics in a manner similar to a likelihood ratio test. To visualize ontogenetic allometric variation, we plotted size against the first principal component (PC) of shape predicted by the full model with unique allometries [8]. We also visualized the trajectories as vectors in morphospace, plotting the first two PCs of predicted shape.

Based on the regressions under the unique-allometry model, we performed interspecific pairwise comparisons of the angles and lengths of the ontogenetic allometric trajectories. Significance was assessed by comparing the empirical values with those obtained through residual randomization. Adjustment of *p* values is not necessary because the residuals are randomly placed in the same way for every test statistic, meaning that each pairwise contrast is a separate comparison derived from the same test [69]. The pairwise comparisons were performed with the “pairwise” and “summary” functions of ‘RRPP’, which also return estimates of the angle and length of the trajectories. We then evaluated whether the species pairs sharing a common slope display overlapping (common intercept) or parallel (differing intercept) trajectories. This was performed with the “int.test” R function [38], which performs a multivariate linear regression of shape on size and compares the Euclidean distances between intercepts to a null distribution obtained through permutation. We performed 10,000 permutations and adjusted *p* to account for type I error.

Finally, we used hierarchical partitioning to estimate the independent and joint effects of differences in trajectory attributes (angle, length, and intercept) on morphological disparity. Briefly, hierarchical partitioning performs multiple regression on all possible combinations of predictors and averages the effect of each of them based on a given goodness-of-fit measure [70]. The joint effect of each predictor in the full model is also extracted from these comparisons [70]. For each species pair, we characterized morphological disparity as the Euclidean distance between the adult phenotypes, disparity in trajectory angles as degrees, disparity in trajectory lengths as the absolute difference between the estimated lengths, and disparity in trajectory intercepts as the Euclidean distance between intercepts. Morphological disparity was obtained from the phenotypes predicted by the unique-allometry model for the largest individuals of each species, disparity in the angles and lengths was obtained from the regressions under the unique-allometry model, and the intercept distances were obtained from the “int.test” analysis. We performed the hierarchical partitioning analyses on the ‘hier.part 1.0.6’ R package [71], based on the Gaussian family function and specifying *R*^2^ as goodness-of-fit measure.

### Heterochrony

In the first approach used to detect heterochrony, we evaluated whether heterochronic changes have contributed to phenotypic differentiation in those species pairs sharing a common slope and intercept (61.07% of species pairs in body dataset, 67.34% in limbs, and 82.6% in head). In the absence of information on age, it is challenging to infer the processes responsible for heterochronic shifts (e.g., progenesis vs. neoteny) [72]. Thus, studies based on wild caught individuals rely on the identification of paedomorphosis and peramorphosis for the detection of heterochrony [10, 38]. To detect paedomorphosis/peramorphosis we performed interspecific pairwise comparisons of the adult morphology, using the distance between the phenotypes at maximum size as test statistic. This was performed with the “peram.test” R function [38], and significance assessed through 10,000 permutations. Pairwise *p* values were adjusted to account for type I error.

For the second approach, we first tested for ontogenetic scaling through the Tfh1 test [14, 73]. In this test, the sum of the squared residuals from the multivariate regression of shape on size is used as test statistic. In species pairs where the hypothesis of ontogenetic scaling was rejected, we tested for heterochrony with size-shape dissociation using the Tfh2 test [14, 73]. In this test, the sum of squared distances from each specimen to its nearest point on the multivariate regression line in shape space is used as test statistic. In both Tfh tests, significance is assessed by randomizing the taxonomic identity of individuals (10,000 permutations for Tfh1 and 500 for Tfh2). To be conservative, in these tests we did not correct for type I error because heterochrony is the null hypothesis.

### Morphological variation in juveniles and adults

We performed the HOST as explained above on Varanidae and each varanid subgenus containing two or more species. For each clade in which the null hypothesis of common slopes was rejected, we tested whether there is evidence for ontogenetic convergence or divergence. The procedure is based on Adams and Nistri [8] and Esquerré et al. [10]. For each species, we obtained the predicted shapes of the largest adult and smallest juvenile from the regressions under the unique-allometry model. We then calculated the pairwise Euclidean distances among juveniles and adults and summed them to obtain a measure of disparity among juveniles (*D*_*j*_) and adults (*D*_*a*_). The test statistic is *D* = *D*_*j*_–*D*_*a*_, which takes a positive value when adults belonging to the different species are more similar to each other than are juveniles (convergence), and a negative value when juveniles are more similar to each other than are adults (divergence). Empirical values of *D* are compared to a distribution obtained by randomizing the morphology of individuals with respect to their size, i.e., picking two random individuals from each species as representatives of the juvenile and adult morphology, respectively. For positive values of *D*, we obtained *p* values by calculating the proportion of permuted values that were larger than or equal to the empirical value. For negative values, we calculated the proportion of permuted values that were smaller than or equal to the empirical value.

We also evaluated the influence of phylogeny, size, and habitat use on juvenile and adult morphology. Our phylogeny is primarily based on a phylogenomic-scale time-calibrated tree [22], trimmed to match our sampling (see Additional file 2: Supporting methods, Fig. S1) (Additional file 1: Table S5). For each species, we obtained the predicted phenotype for the largest adult and smallest juvenile from the unique-allometry regressions. We estimated phylogenetic signal as the multivariate version of Blomberg’s *K* [35, 36] for each morphological dataset and growth stage employing the “physignal” function of ‘geomorph’. We evaluated the significance of the phylogenetic signal through 10,000 permutations. To assess whether phylogenetic signal differs significantly between growth stages we calculated Δ*K* = adult *K* (*K*_*a*_)–juvenile *K* (*K*_*j*_). We obtained a null distribution of Δ*K* by randomizing the morphology of individuals with respect to their size, i.e., picking two random individuals from each species as representatives of the juvenile and adult morphology, respectively. We obtained *p* values by calculating the proportion of permuted values that were larger than or equal to the empirical value.

We evaluated the influence of size, habitat use, and their interaction on juvenile and adult shape through a multivariate approach based on the phylogenetic ANOVA of Garland et al. [74], hereafter referred to as phylogenetic MANOVA. The shapes predicted for the largest and smallest specimens of each species were specified as adult and juvenile shape, respectively. In the case of adults, size was specified as log-transformed maximum snout-vent-length (SVL; commonly used as a proxy of body size in reptiles), which was obtained from the literature or our own specimen examination (Additional file 1: Table S1). In the case of juveniles, we employed log-transformed minimum SVL, which was obtained from our sample. Based on natural history literature (Additional file 1: Table S1), we classified each species and growth stage independently into six habitat use categories: amphibious (semiaquatic species), canopy (arboreal species that use narrow branches high in trees), cryptic (species that spend considerable time under cover), escarpment (species that move vertically on rocky cliffs), terrestrial (species that move extensively through open habitats), and trunk (arboreal species that use the wider limbs of trees). In our procedure, we first performed principal component analysis (PCA) on the shapes of adults and juveniles and retained the first PCs that cumulatively account for 95% of variance or more. We then fitted a linear model using the “procD.lm” function of ‘geomorph’, with the PC scores as response variables and size, habitat, and their interaction as predictors. Next, we fitted a Brownian motion model on the PC scores in ‘mvmorph 1.1.1’ [75] and simulated 10,000 datasets under the estimated parameters to obtain a null distribution of *F* statistics. Finally, we compared the empirical *F* statistics with the null distribution to obtain a *p* value. Additionally, to visualize the phylogenetic and ecological influence on morphology we plotted the phylomorphospace of juveniles and adults. We plotted the first two PCs, colored each species according to its subgenus or ecology, and overlaid the phylogeny on the plot using the ‘phytools 0.7.62’ R package [76].

### Evolution of trajectories

We estimated the phylogenetic signal and evaluated the influence of adult size and habitat use in the length and slope of the ontogenetic allometric trajectories obtained from the regressions under the unique-allometry model. We calculated Blomberg’s *K* and assessed its significance using the “physignal” function. We then used the phylogenetic MANOVA to evaluate whether trajectory attributes are influenced by adult size, habitat use, and their interaction. In the case of the slopes, it was necessary to reduce dimensionality by keeping the first PCs that account for 95% of variance or more. We specified the log-transformed maximum SVL as proxy for adult size and classified the species that experience ontogenetic shifts in habitat use in a different category to either the juvenile or adult ecology; e.g., those species that are trunk dwellers as juveniles and become terrestrial as adults were grouped together and separate from those that are either trunk dwellers or terrestrial throughout their lives. Additionally, we visualized allometric diversity in the group by plotting the phyloallomspace [10].

To infer the evolutionary mode of the ontogenetic allometric trajectories, we fitted evolutionary models to the trajectory lengths and first PC of the slopes. For some analyses requiring it, we obtained stochastic maps for habitat use and biogeographic history (Additional file 2: Supporting methods, Figs. S13, S14). We used the R packages ‘geiger 2.0.6.4’ [77], ‘mvmorph 1.1.1’ [75], and ‘RPANDA 1.7’ [78] to fit eight models: (1) Brownian motion (BM); (2) BM with different parameters for each habitat use category (BMS) (non-censored approach [79]); (3) Ornstein–Uhlenbeck model (OU); (4) OU with multiple optima, one for each habitat use category (OUM); (5) early burst model (EB); (6) matching-competition model (MC); (7) linear diversity dependent (DDl); and (8) exponential diversity dependent (DD2). We limited competition in the last three models to those taxa occurring in sympatry. We compared the models based on the sample-size-corrected Akaike information criterion (AICc) and respective weights (AICcw).

To examine the rates of evolution of the trajectory attributes (length and slope), we estimated branch-specific rates of evolution based on phylogenetic ridge regression in ‘RRphylo 2.4.7’ [80]. We specified the trajectory lengths themselves as covariates so that rates are not artificially inflated for species experiencing large magnitudes of ontogenetic change [80]. To visualize rate variation across the phylogeny, we log-transformed the absolute rates, because ‘RRphylo’ indicates the direction of phenotypic change by labeling shifts as positive or negative. We used the “search.shift” function of ‘RRphylo’ to detect rate shifts. This function calculates the difference between background rates and each clade containing a minimum number of species (specified by the user; six in this case). Clade-specific rates are then compared against this null distribution of rate differences to detect shifts (*p* > 0.975 indicates significantly higher rates and *p* < 0.025 indicates significantly lower rates) [80]. We evaluated the sensitivity of our results to taxon sampling and phylogenetic uncertainty using the “overfitRR” function, which iteratively removes and rearranges tips [80]. We specified 100 tree-modification iterations removing 25% of tips and modifying the position and age of 25% of tips and nodes, respectively. Nodes with a posterior probability above 0.95 were forced to remain monophyletic.

## Supplementary Information

**Additional file 1: Table S1.** Species included in this study and associated metadata. **Table S2.** Linear morphometric data describing body shape (in mm). **Table S3.** Linear morphometric data describing limb shape (in mm). **Table S4.** Procrustes aligned coordinates of head shape. **Table S5.** GenBank accession numbers (GB) for molecular data used in phylogenetic analyses of *Euprepiosaurus* and *Hapturosaurus*. **Table S6.** Results of the isometry test. **Table S7.** Results of the homogeneity of slopes tests. **Table S8.** Regression parameters under the unique-allometry model. **Table S9.** Ontogenetic change in examined characters. **Table S10.** Estimated lengths of ontogenetic allometric trajectories. **Table S11.** Estimated angles of ontogenetic allometric trajectories. **Table S12.** Pairwise comparison of the lengths of the ontogenetic allometric trajectories (body). **Table S13.** Pairwise comparison of the lengths of the ontogenetic allometric trajectories (limbs). **Table S14.** Pairwise comparison of the lengths of the ontogenetic allometric trajectories (head). **Table S15.** Pairwise comparison of the slopes of the ontogenetic allometric trajectories (body). **Table S16.** Pairwise comparison of the slopes of the ontogenetic allometric trajectories (limbs). **Table S17.** Pairwise comparison of the slopes of the ontogenetic allometric trajectories (head). **Table S18.** Pairwise comparison of the intercepts of the ontogenetic allometric trajectories (body). **Table S19.** Pairwise comparison of the intercepts of the ontogenetic allometric trajectories (limbs). **Table S20.** Pairwise comparison of the intercepts of the ontogenetic allometric trajectories (head). **Table S21.** Goodness of fit measures used in the hierarchical partitioning analyses. **Table S22.** Results of hierarchical partitioning analyses. **Table S23.** Peramorphosis test (body). **Table S24.** Peramorphosis test (limbs). **Table S25.** Peramorphosis test (head). **Table S26.** Size-shape space overlap test of heterochrony (Tfh1) (body). **Table S27.** Size-shape space overlap test of heterochrony (Tfh1) (limbs). **Table S28.** Size-shape space overlap test of heterochrony (Tfh1) (head). **Table S29.** Shape space overlap test of heterochrony (Tfh2) (body). **Table S30.** Shape space overlap test of heterochrony (Tfh2) (limbs). **Table S31.** Shape space overlap test of heterochrony (Tfh2) (head). **Table S32.** Results of the phylogenetic signal tests. **Table S33.** Results of the phylogenetic MANOVA of morphological/ontogenetic data against size, habitat, and their interaction. **Table S34.** Results of evolutionary model fitting on the length of ontogenetic allometric trajectories. **Table S35.** Parameter estimates of best-fitting evolutionary models. **Table S36.** Results of evolutionary model fitting on the angle of ontogenetic allometric trajectories.

**Additional file 2:** Supporting methods. **Figure S1.** Time-calibrated phylogeny of Paleoanguimorpha from Pavón-Vázquez et al. (in press). **Figure S2.** Ontogenetic change in body shape with size. **Figure S3.** Ontogenetic change in limb shape with size. **Figure S4.** Ontogenetic change in head shape with size. **Figure S5.** Ontogenetic change of body shape in morphospace. **Figure S6.** Ontogenetic change of limb shape in morphospace. **Figure S7.** Ontogenetic change of head shape in morphospace. **Figure S8.** Ontogenetic allometric trajectories colored by habitat use. **Figure S9.** Morphological and ontogenetic disparity. **Figure S10.** Relationship between body size and the magnitude of ontogenetic shape change. **Figure S11.** Phylomorphospace of juvenile and adult paleoanguimorphs. **Figure S12.** Phyloallomspace of Paleoanguimorpha. **Figure S13.** Stochastic character mapping of habitat use in Paleoanguimorpha. **Figure S14.** Ancestral range reconstruction of Paleoanguimorpha from Pavón-Vázquez et al. (in press). **Supporting references.**

## Data Availability

The morphological datasets supporting the conclusions of this article are included within the additional files. The molecular datasets are available in the Dryad Digital Repository, at https://dx.doi.org/10.5061/dryad.tx95x69t8 and https://doi.org/10.5061/dryad.m0n61, and in the National Center for Biotechnology Information (see details in Additional file 1: Table S5).
