# Supplementary material for: Ontogenetic drivers of morphological evolution in monitor lizards and allies (Squamata: Paleoanguimorpha), a clade with extreme body size disparity
Source: BMC Ecol Evol. 2022 Feb 12;22:15. doi: 10.1186/s12862-022-01970-6 (PMC8840268; doi:10.1186/s12862-022-01970-6)
Supplement: Supplementary file 2 — Additional file 2: Supporting methods. Figure S1. Time-calibrated phylogeny of Paleoanguimorpha from Pavón-Vázquez et al. (in press). Figure S2. Ontogenetic change in body shape with size. Figure S3. Ontogenetic change in limb shape with size. Figure S4. Ontogenetic change in head shape with size. Figure S5. Ontogenetic change of body shape in morphospace. Figure S6. Ontogenetic change of limb shape in morphospace. Figure S7. Ontogenetic change of head shape in morphospace. Figure S8. Ontogenetic allometric trajectories colored by habitat use. Figure S9. Morphological and ontogenetic disparity. Figure S10. Relationship between body size and the magnitude of ontogenetic shape change. Figure S11. Phylomorphospace of juvenile and adult paleoanguimorphs. Figure S12. Phyloallomspace of Paleoanguimorpha. Figure S13. Stochastic character mapping of habitat use in Paleoanguimorpha. Figure S14. Ancestral range reconstruction of Paleoanguimorpha from Pavón-Vázquez et al. (in press). Supporting references. [file 12862_2022_1970_MOESM2_ESM.docx]

**ADDITIONAL FILE 2**

**Supporting Methods**

The taxonomic framework, phylogeny, morphological data processing, and ancestral range reconstruction are based on an unpublished study on the diversification of Varanidae (Pavón-Vázquez et al., in press). The phylogeny was trimmed to match our sampling.

**Taxonomic background**

*Varanus* *griseus* *caspius* was treated as a full species by Brennan et al. (2021), but pending a more rigorous study on the widespread and polytypic *V*. *griseus* here we consider it as a single evolutionary unit. Eidenmüller et al. (2017) showed that *V*. *prasinus* is polyphyletic. Thus, we consider the samples from the vicinities of the type locality of western Papua New Guinea as true *V*. *prasinus*, including *V*. *reisingeri* which was recently relegated as a subspecies of *V*. *prasinus* (Bucklitsch et al. 2016), and excluded other samples of *V*. *prasinus* from our sampling pending a more rigorous taxonomic assessment of the group. Populations in the *V*. *indicus* complex from the Solomon Islands apparently represent an undescribed lineage (Weijola et al. 2019). While additional specific diversity may be present within the lineage (Weijola et al. 2019), in this study we conservatively consider it as a single species (*Varanus* sp. Solomon Islands). Three species closely related to *V*. *chlorostigma* have been recently described or resurrected: *V*. *bennetti*, *V*. *colei*, and *V*. *tsukamotoi* (Böhme et al. 2019; Weijola et al. 2020). Divergence of these species from topotypic *V*. *chlorostigma* is fairly recent (< 2 Ma) (Weijola et al. 2019), and their recognition would force us to acknowledge other populations currently allocated to *V*. *chlorostigma* as separate species. Thus, for the purposes of this study, we have analyzed *V*. *bennetti*, *V*. *colei*, *V*. *chlorostigma*, and *V*. *tsukamotoi* as a single evolutionary unit (*V*. *chlorostigma*). Species limits in the Australo-Papuan populations of the *V*. *timorensis* complex are ill-defined, and we have considered all of them under *V*. *scalaris* (Smith et al. 2004). The subspecies *V*. *acanthurus* *insulanicus* is consistently recovered as sister to *V*. *baritji* and is apparently more distantly related to other populations of *V*. *acanthurus* (Fitch et al. 2006; Brennan et al. 2021; Pavón-Vázquez et al. in prep.). Thus, we excluded individuals of *V*. *a*. *insulanicus* from our sample of *V*. *acanthurus*.

**Morphological data**

The linear measurements that we recorded to characterize body shape were: head length (along dorsal midline, between tip of snout and anterior edge of tympanum), head width (at level of anterior edge of tympanum), head depth (at level of middle of eyes), neck length (between anterior edge of tympanum and gular fold), body length (between gular fold and vent), hip width (width of pelvic gridle measured at level of middle of hindlimbs), tail length (between vent and tip of tail), tail width (measured at level of one third of tail length from vent), and tail depth (measured at level of one third of tail length from vent) (Additional file 2: Fig. 2). The measurements used to characterize limb shape were: upper arm length (between base of forelimb and elbow), lower arm length (between elbow and wrist), hand length (between wrist and base of finger IV), hand width (perpendicular to base of finger V), finger IV length (between base of finger and proximal edge of claw), upper leg length (between base of hindlimb and knee), lower leg length (between knee and ankle), foot length (between ankle and base of toe IV), foot width (perpendicular to base of toe V), and toe IV length (between base of toe and proximal edge of claw) (Additional file 2: Fig. 2). The landmarks describing head shape are: tip of snout, anterior edges of supraocular semicircles, medial edges of supraocular semicircles, posterior edges of supraocular semicircles, anterior edges of tympanum, posterior edges of nuchal fold, and anterior edge of nuchal fold (Additional file 2: Fig. 2). Ten sliding semi-landmarks were placed on each side between the tip of the snout and the anterior edge of the tympanum (Additional file 2: Fig. 2). *Lanthanotus* lacks an external tympanum, but a depression covered with scales smaller than surrounding osteoderms indicates the position of the auricular organs (Maisano et al. 2002).

We imputed missing data (i.e., from specimens having incomplete tails and/or toes) using random forest training in ‘missForest 1.4’ (Stekhoven and Bühlmann 2012), including species and sex as predictors. To account for relatedness in data imputation, we performed the procedure separately for *Shinisaurus*, *Lanthanotus*, and each varanid subgenus. Due to practical reasons, large specimens of larger species are rare in collections. Therefore, the mean body size in our sample may not be representative of body size variation in the wild and there is systematic bias towards smaller sizes in larger species compared to smaller species. Furthermore, reptiles show indeterminate growth and thus snout-vent-length (SVL) is correlated with age, and thus some authors advocate for the use of maximum SVL in reptile comparative studies (e.g. Stamps and Andrews 1992; Greer 2001; Sherratt et al. 2018). Thus, we based our analyses on maximum SVL instead of mean SVL. We tested for sexual dimorphism in the linear measurements for each species in which each sex was represented by at least three specimens. We compared the sexes through an analysis of variance using the ‘procD.lm’ function of the ‘geomorph 3.0.3’ R package (Adams and Otarola-Castillo 2013), assessing significance by performing 1,000 permutations. Since our sampling is biased towards males, we discarded females for those species in which sexual dimorphism was significant at the 95% confidence level.

When taking the head photographs, we tried to keep position and orientation as consistent as possible. Digitalization and processing of the landmark data was performed in ‘geomorph 3.0.3’ (Adams and Otarola-Castillo 2013). In damaged specimens, we estimated the position of missing landmarks per species using the thin-spline method (Gunz et al. 2009). Same as for the linear measurements, we tested for sexual dimorphism in the Procrustes aligned coordinates and removed females of the sexually dimorphic species. After removing females of the sexually dimorphic species, we repeated the generalized Procrustes analysis.

**Phylogenetics**

The tree is mainly based on the extant-only phylogeny of varanids presented by Brennan et al. (2021). This phylogeny was based on 60 nuclear exons obtained through anchored hybrid enrichment (Lemmon et al. 2012). We based the phylogenetic position and divergence dates of *Lanthanotus* *borneensis*, *V*. *dumerilii*, and *V*. *nebulosus* on another recently published multi-locus phylogeny (Lin and Wiens 2017). The most complete phylogenies of the *Euprepiosaurus* and *Hapturosaurus* subgenera and the *salvator* complex in the *Soterosaurus* subgenus have been published independently from each other and some of the relationships and/or divergence dates are in conflict with those of Brennan et al. (2021). Thus, we estimated a time-calibrated phylogeny for each of these subgenera using available molecular data and based our dating on the phylogeny of Brennan et al. (2021), subsequently binding the trees to our backbone phylogeny. All analyses were conducted in BEAST 2.5.1 (Bouckaert et al. 2014).

The phylogeny of *Euprepiosaurus* was based on sequences of the *16S* and *ND4* mitochondrial loci (Additional file 1: Table S5). We assigned each locus to its own partition and selected substitution models based on the Bayesian information criterion using ModelFinder (Kalyaanamoorthy et al. 2017). The best-fitting models were TIM3e+I (*16S*) and TN+F+G4 (*ND4*). We built a species tree specifying a relaxed log-normal clock model and a Yule tree model. We included *V*. *prasinus* as outgroup and specified a normal distribution for the root-age prior (*x̄* = 13.02, σ = 0.001). We ran two independent analyses for 1,000 million generations with sampling every 25,000 iterations. We verified convergence and adequate sampling for every parameter (effective sample size > 200), combined the runs, deleted 10% of samples as burnin, and extracted the maximum clade credibility (MCC) tree with mean branch lengths.

The phylogeny of *Hapturosaurus* was based on unique haplotypes of *16S* (Additional file 1: Table S5). The sequence of *V*. *bogerti* comes from Eidenmüller et al. (2017). The best-fitting model was TIM2e+I. We specified a relaxed log-normal clock model and a Yule tree model. We included *V*. *chlorostigma* as outgroup and specified a normal distribution for the root-age prior (*x̄* = 13.02, σ = 0.001). We ran two independent analyses for 100 million generations with sampling every 5,000 iterations and obtained the MCC tree. For downstream analyses, we only kept one individual per species (all species were monophyletic).

Finally, the phylogeny of the *salvator* complex was based on 4 nuclear loci (*PRLR* and anonymous nuclear loci L44, L52, and L74) and a mitochondrial fragment containing *ND1*, *ND2*, and associated tRNAs (available at https://doi.org/10.5061/dryad.m0n61). The best-fitting models were F81+F (L44), HKY+F+I (L52), and JC (L74 and *PRLR*). Due to mixing problems, we chose a relatively simple model for the mitochondrial fragment (HKY+F) and specified a strict molecular clock. We built a species tree based on the Yule model. We included *V*. *rudicollis* as outgroup and specified a normal distribution for the root-age prior (*x̄* = 5.95, σ = 0.001). We ran two independent analyses for 1,000 million generations with sampling every 50,000 iterations and obtained the MCC tree.

**Stochastic mapping of habitat use**

We reconstructed the evolution of habitat use in Paleoanguimorpha through stochastic character mapping based on maximum likelihood. We used the “make.simmap” function of ‘phytools 0.7.62’ (Revell 2012) and specified a model with equal rates of transition between states. We obtained 1,000 stochastic maps and randomly selected one stochastic map for the model-fitting analyses.

**Biogeography**

We limited competition to sympatric taxa when fitting the matching competition and diversity dependent models. This required us to reconstruct the biogeographic history of Paleoanguimorpha. We used the maximum likelihood implementation of the ‘BioGeoBEARS 1.1.2’ (Matzke 2013) R package. We divided the range of paleoanguimorphs into seven biogeographic regions, primarily based on the zoogeographic realms of Holt et al. (2013). These lizards are only marginally distributed in the Palearctic and Sino-Japanese realms, and the species present there are more widely distributed in the Saharo-Arabian and Oriental realms, respectively. Thus, we considered the former realms as part of the latter. We considered the Philippines east of Huxley’s Line as a separate biogeographic unit given their oceanic origin (Hall 1996, 1998) and high levels of endemicity, including varanids in the *Philippinosaurus* and *Soterosaurus* subgenera. We also considered the islands of eastern Melanesia (including the Solomon Islands and islands of northeastern Papua New Guinea) as a separate unit, given their biogeographic uniqueness as part of the Vitiaz Arc (Ewart 1988; Lucky and Sarnat 2010), including the presence of endemic varanids in the *Euprepiosaurus* and *Solomonsaurus* subgenera.

We tested three main biogeographic models (Matzke 2014): Dispersal-Extinction-Cladogenesis (DEC) (Ree and Smith 2008), and the likelihood implementations of the Dispersal-Vicariance Analysis (DIVALIKE) (Ronquist 1997) and BayArea models (BAYAREALIKE) (Landis et al. 2013). In addition to the basic implementation, for each of the models we incorporated free parameters corresponding to founder-event speciation (*j*), dispersal probability as a function of distance (*x*), and both *j* and *x*, resulting in a total of 12 models. We accounted for plate tectonics and island surfacing by performing a time-stratified analysis with 14 time slices: 2.5 Ma, 5–40 Ma with 5 Ma increases, and 60–140 Ma with 20 Ma increases. To estimate *x*, we calculated the minimum distance between regions for each time slice using GPlates 2.2.0 (Müller et al. 2018). Additionally, we incorporated matrices specifying which areas are allowed at each time slice. Insular regions were only allowed after their time of emergence. Areas incorporating two or more regions were only allowed when the distance between each of their components was below the median. We considered the model with the lowest sample-size corrected Akaike Information Criterion (AICc) as the preferred model, and then compared it against nested models using likelihood ratio tests (LRT). The model with the lowest AICc was DEC+*j*+*x*, and LRT also favored this model (*p* against DEC, DEC+*j*, and DEC+*x* < 0.001). We obtained 50 stochastic maps from the reconstruction based on the preferred model and randomly selected one stochastic map for the model-fitting analyses.

**Supporting Figures**

**
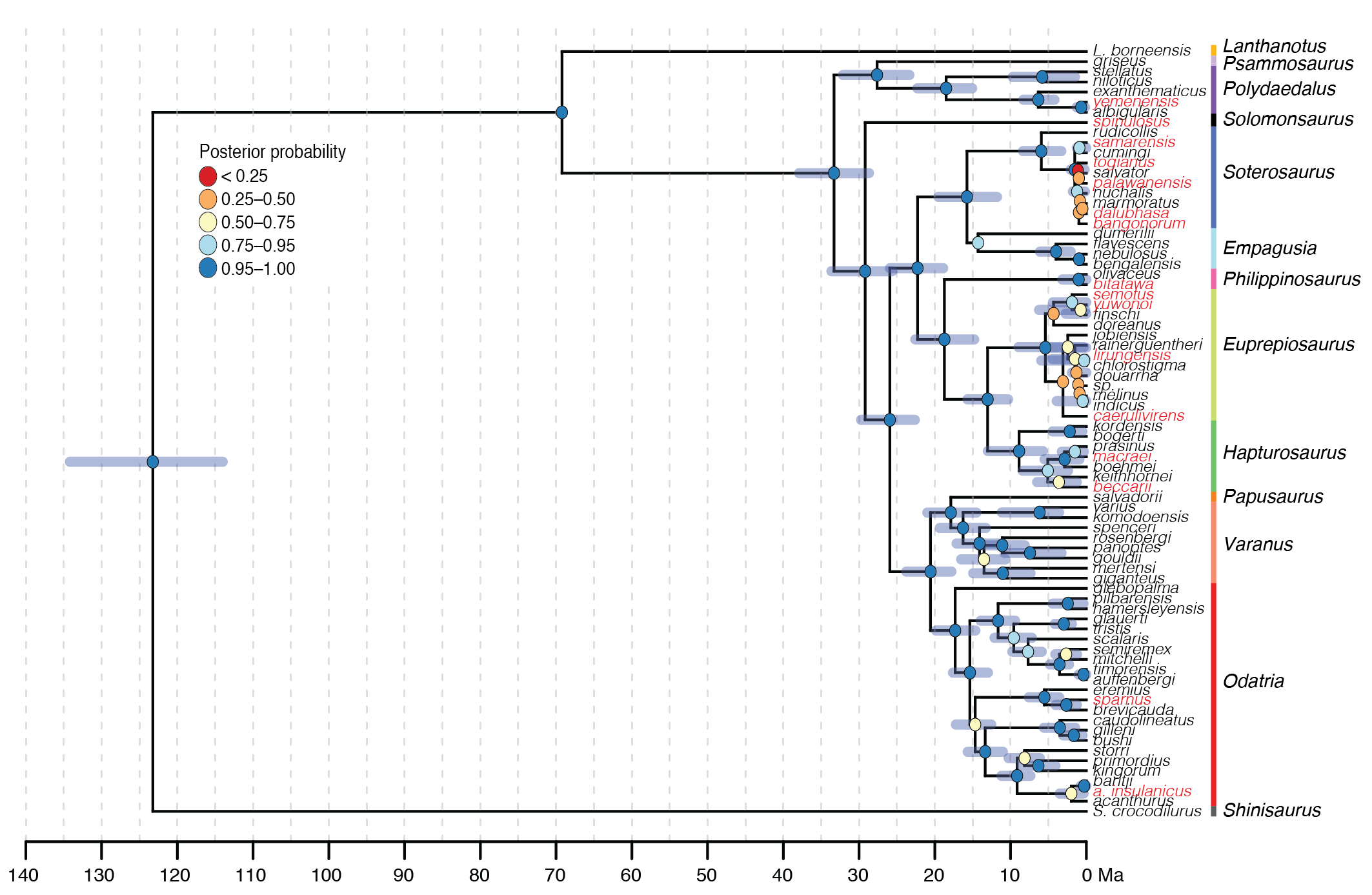
**

**Figure S1.** Time-calibrated phylogeny of Paleoanguimorpha from Pavón-Vázquez et al. (in press). Nodes are colored based on their posterior probability. Blue bars denote the 95% highest posterior density of divergence times (not shown for sections of the tree based on Lin and Wiens (2017) and nodes with posterior probability < 0.50). Taxa in red were excluded from the ontogenetic analyses.


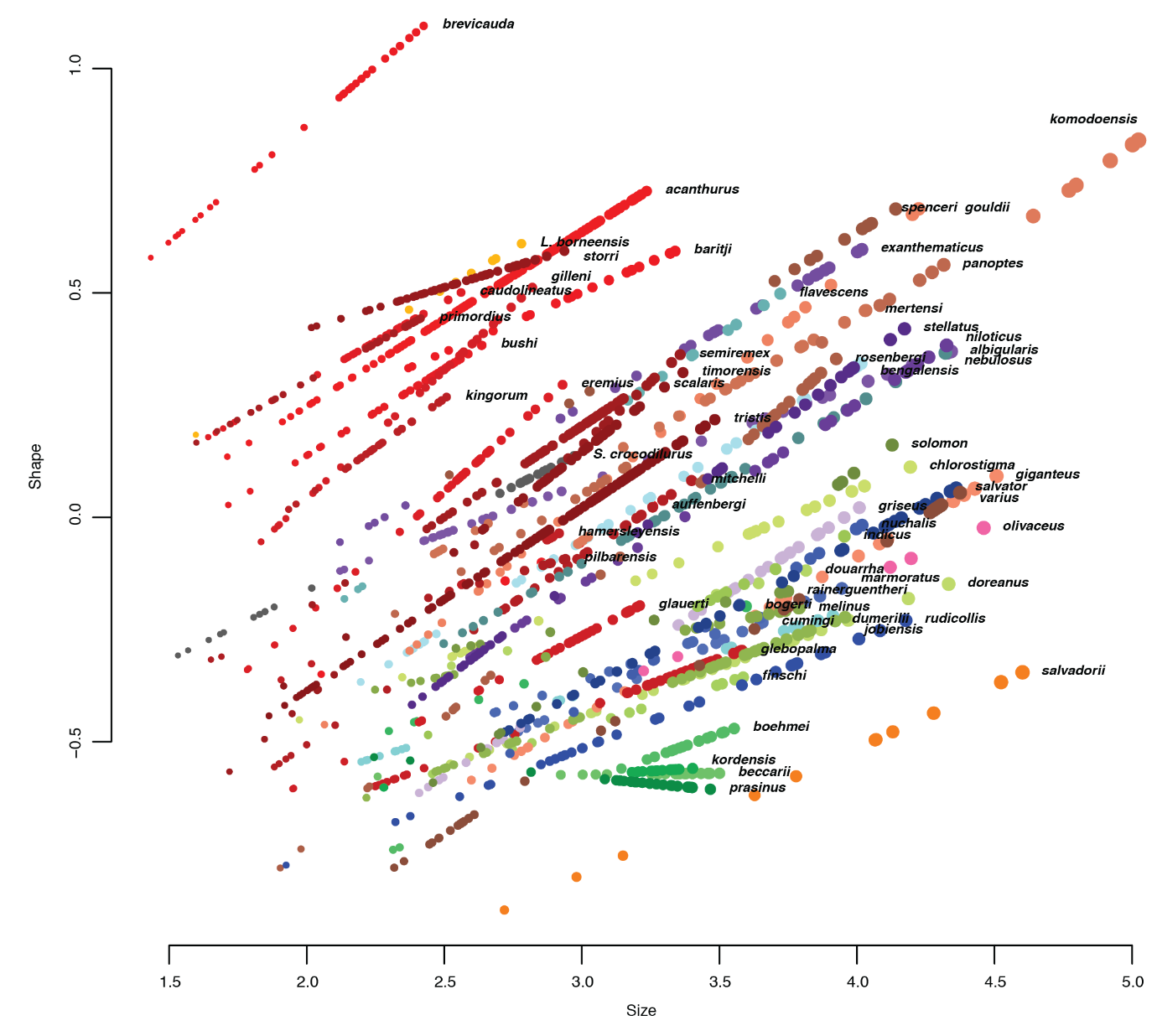


**Figure S2.** Ontogenetic change in body shape with size. The horizontal axis represents size (log-transformed geometric mean of linear measurements) and the vertical axes the first principal component of the predicted shape. Points represent individuals. Similar colors are used for species belonging to the same genus/subgenus.


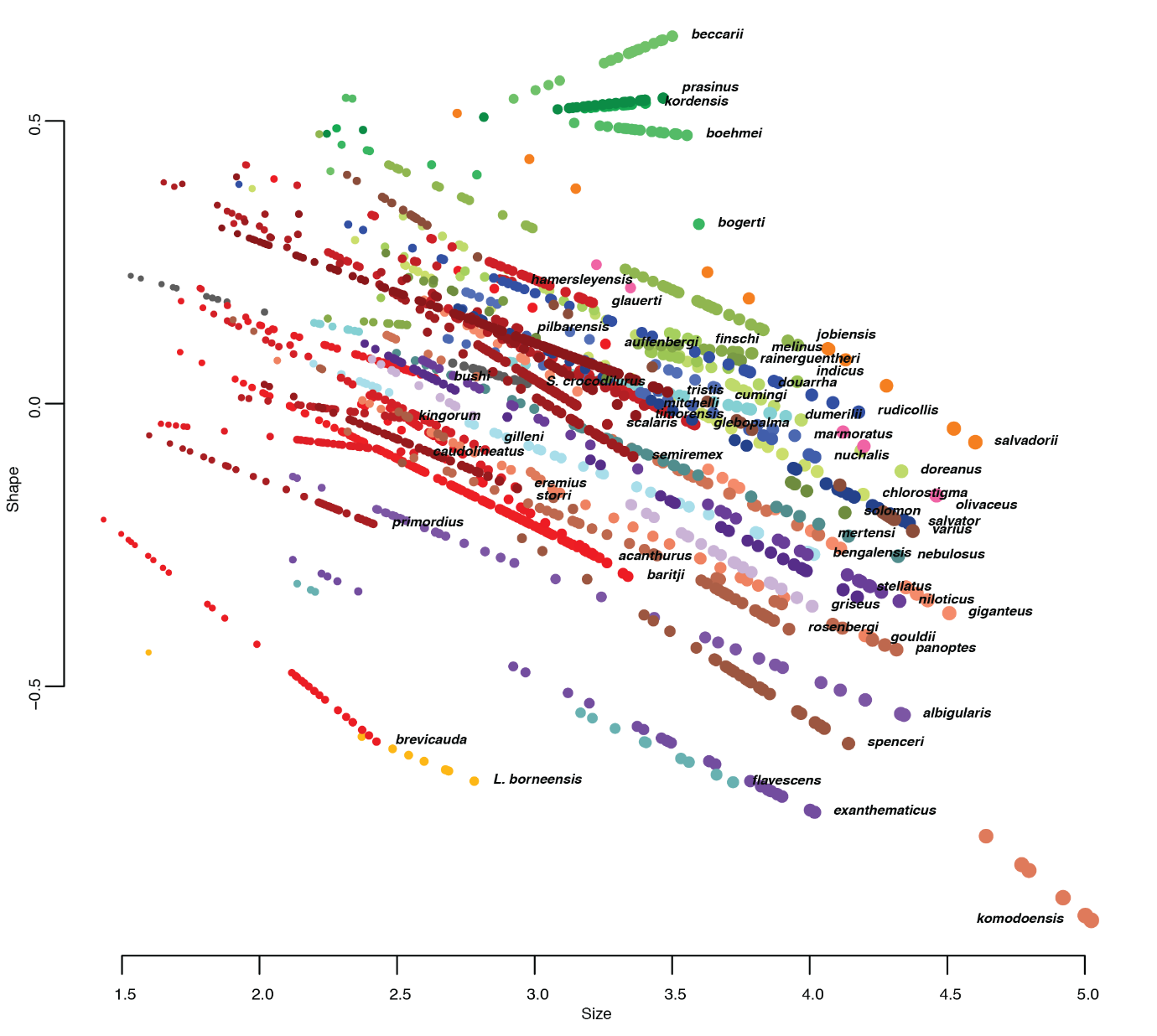


**Figure S3.** Ontogenetic change in limb shape with size. The horizontal axis represents size (log-transformed geometric mean of linear measurements) and the vertical axes the first principal component of the predicted shape. Points represent individuals. Similar colors are used for species belonging to the same genus/subgenus.


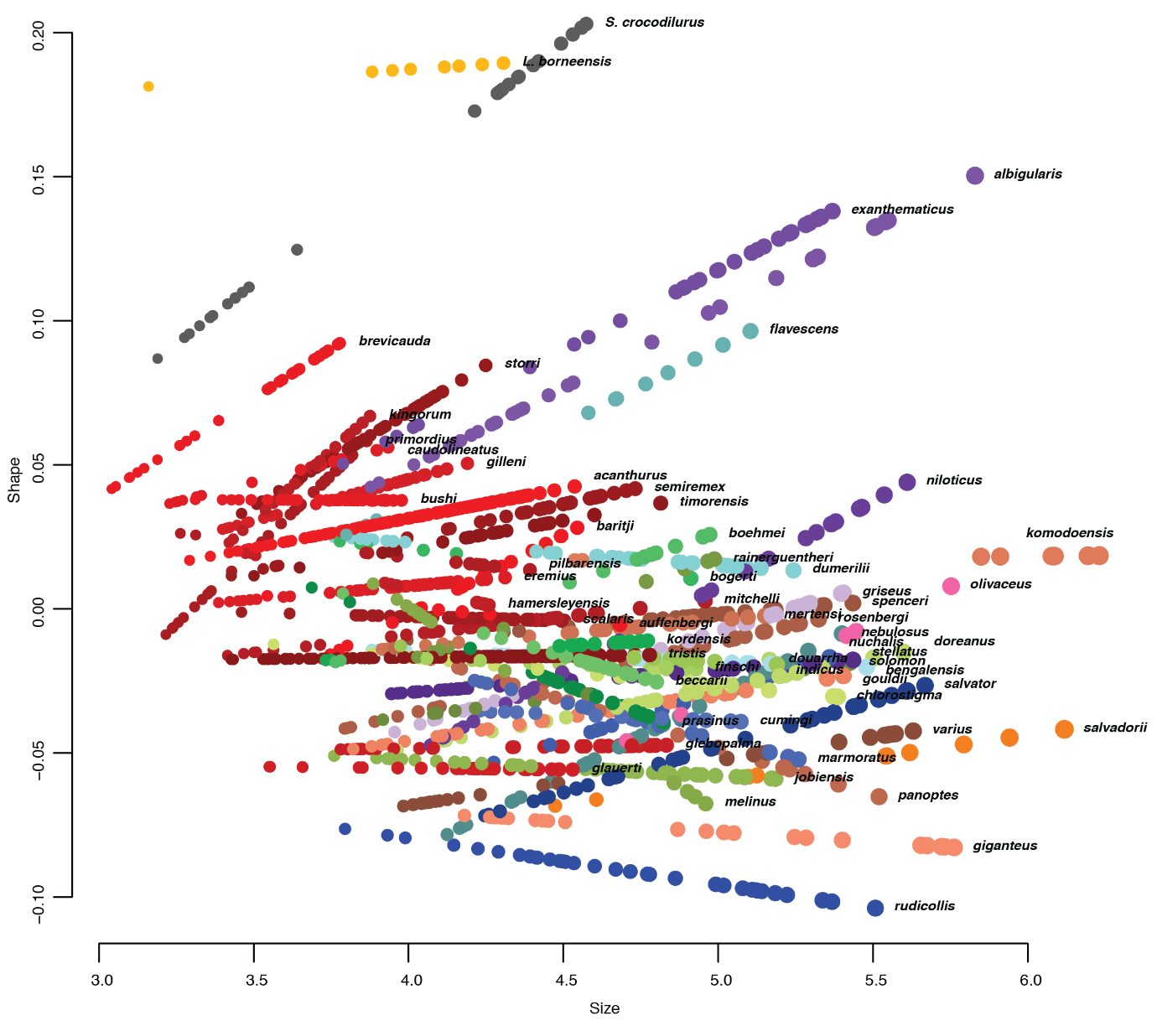


**Figure S4.** Ontogenetic change in head shape with size. The horizontal axis represents size (log-transformed centroid size) and the vertical axes the first principal component of the predicted shape. Points represent individuals. Similar colors are used for species belonging to the same genus/subgenus.


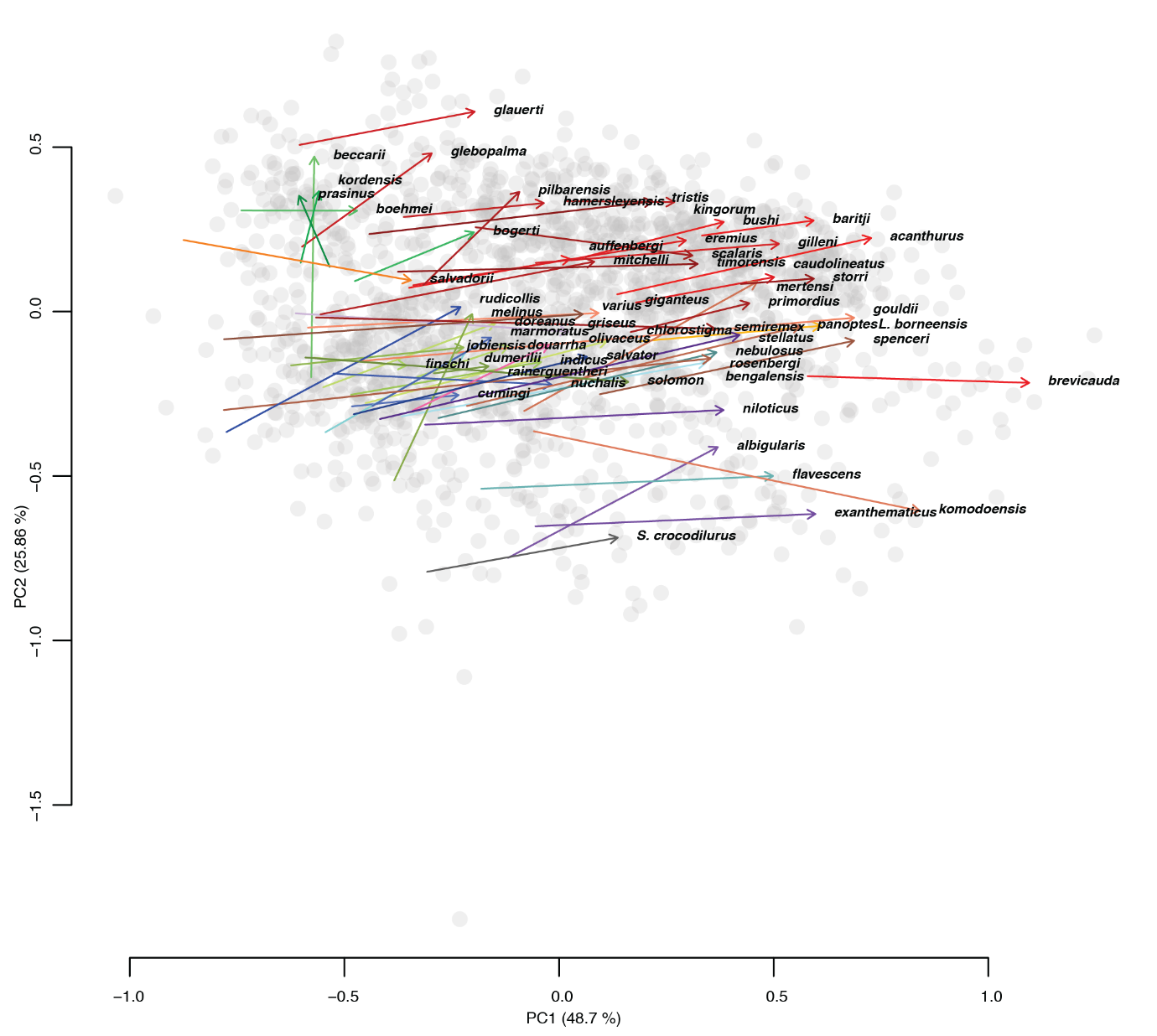


**Figure S5.** Ontogenetic change of body shape in morphospace. The horizontal and vertical axes represent the first and second principal components of the variables describing predicted shape, respectively. Similar colors are used for species belonging to the same genus/subgenus. Gray points represent individuals.


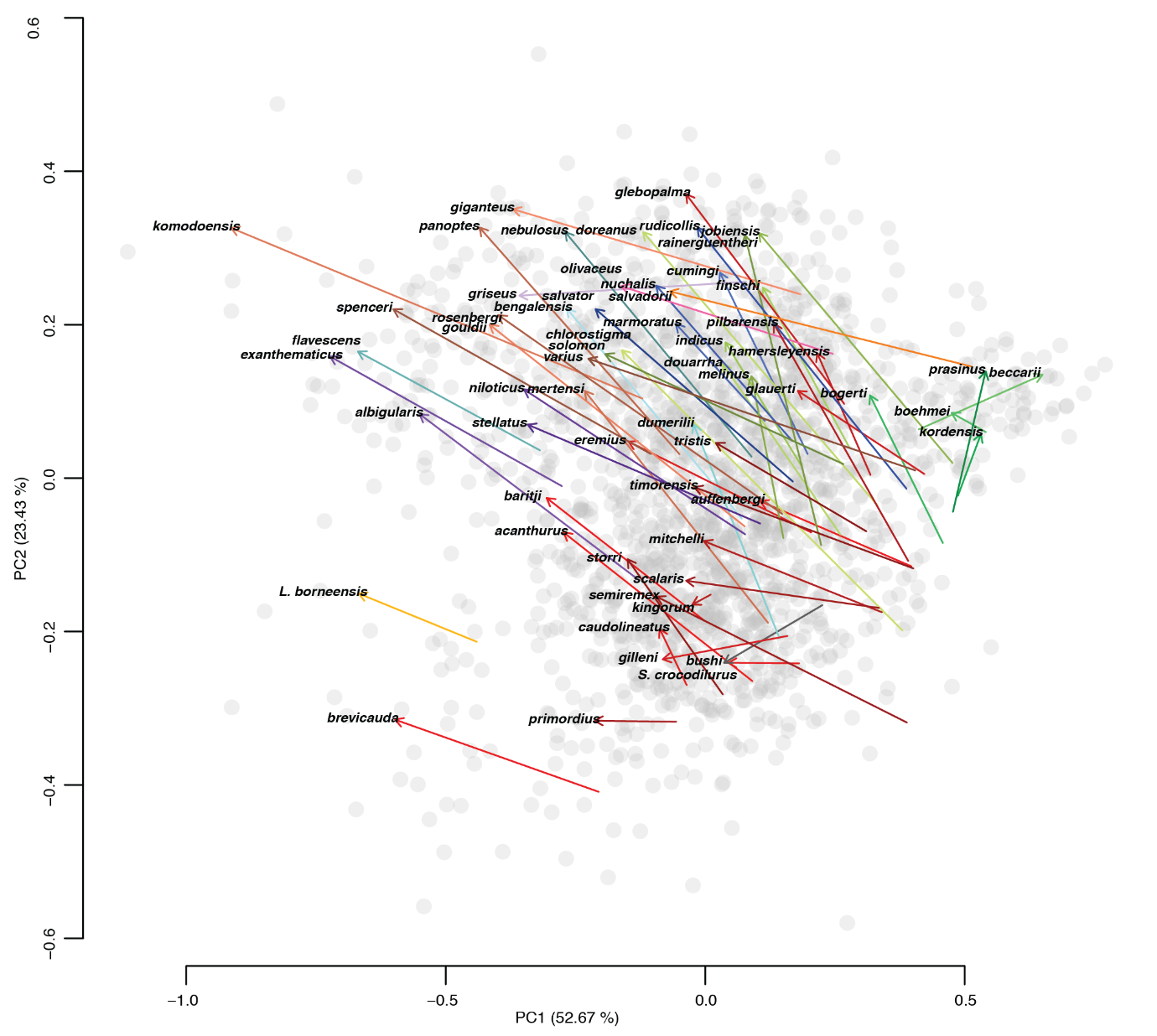


**Figure S6.** Ontogenetic change of limb shape in morphospace. The horizontal and vertical axes represent the first and second principal components of the variables describing predicted shape, respectively. Similar colors are used for species belonging to the same genus/subgenus. Gray points represent individuals.


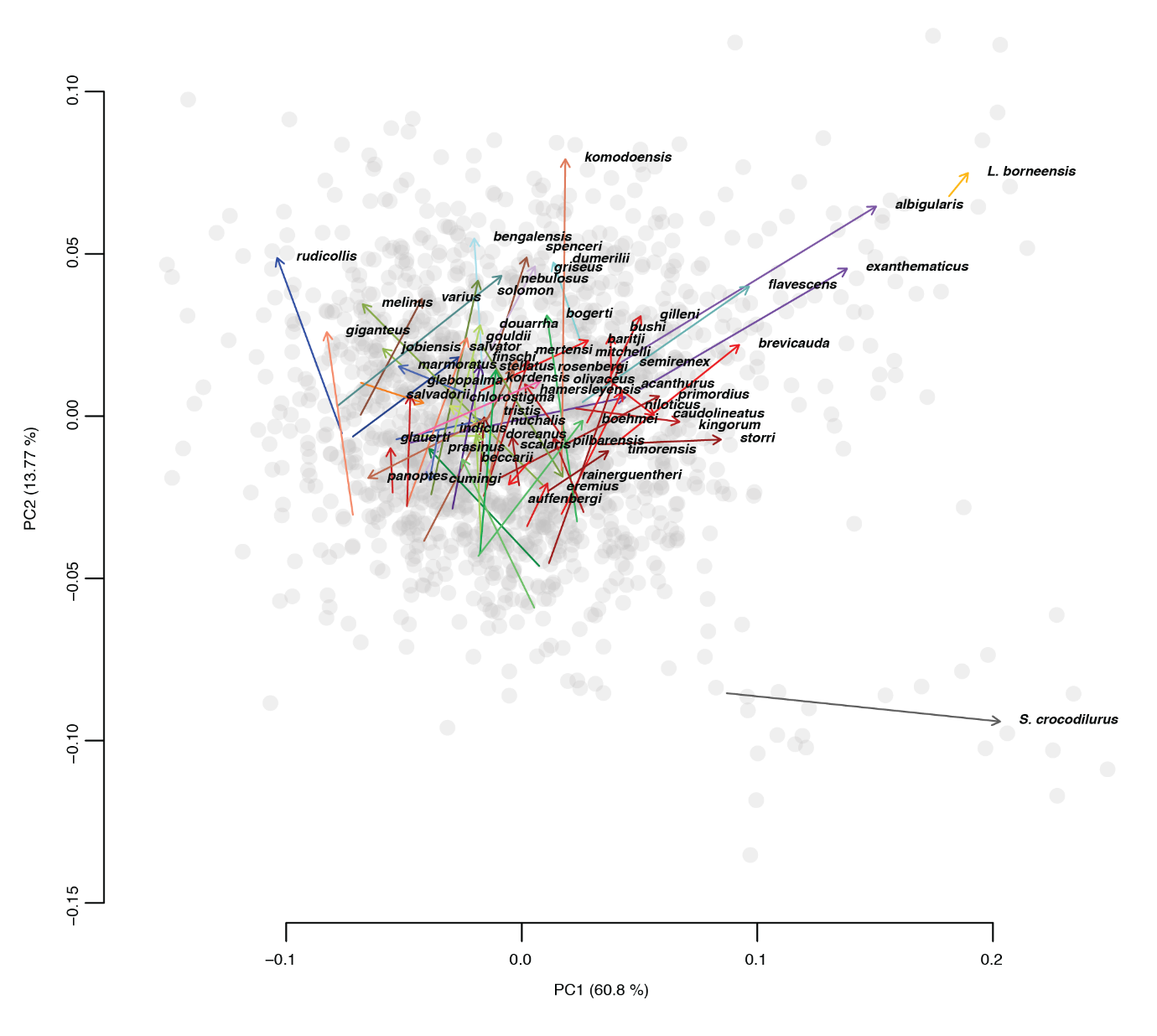


**Figure S7.** Ontogenetic change of head shape in morphospace. The horizontal and vertical axes represent the first and second principal components of the variables describing predicted shape, respectively. Similar colors are used for species belonging to the same genus/subgenus. Gray points represent individuals.


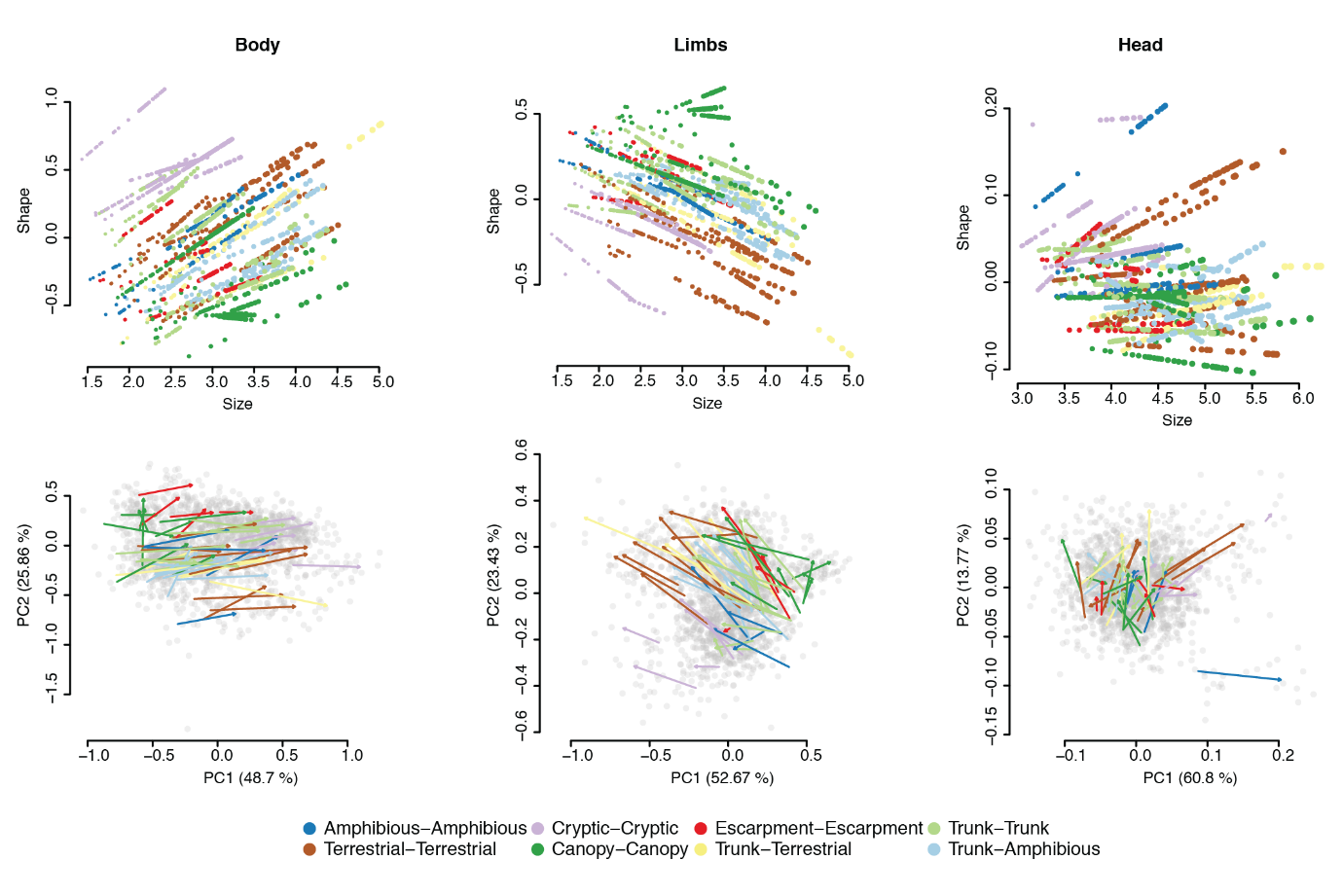


**Figure S8.** Ontogenetic allometric trajectories colored by habitat use. Ontogenetic change in the predicted shape is shown, both with respect to size (top row) and in morphospace (bottom row).


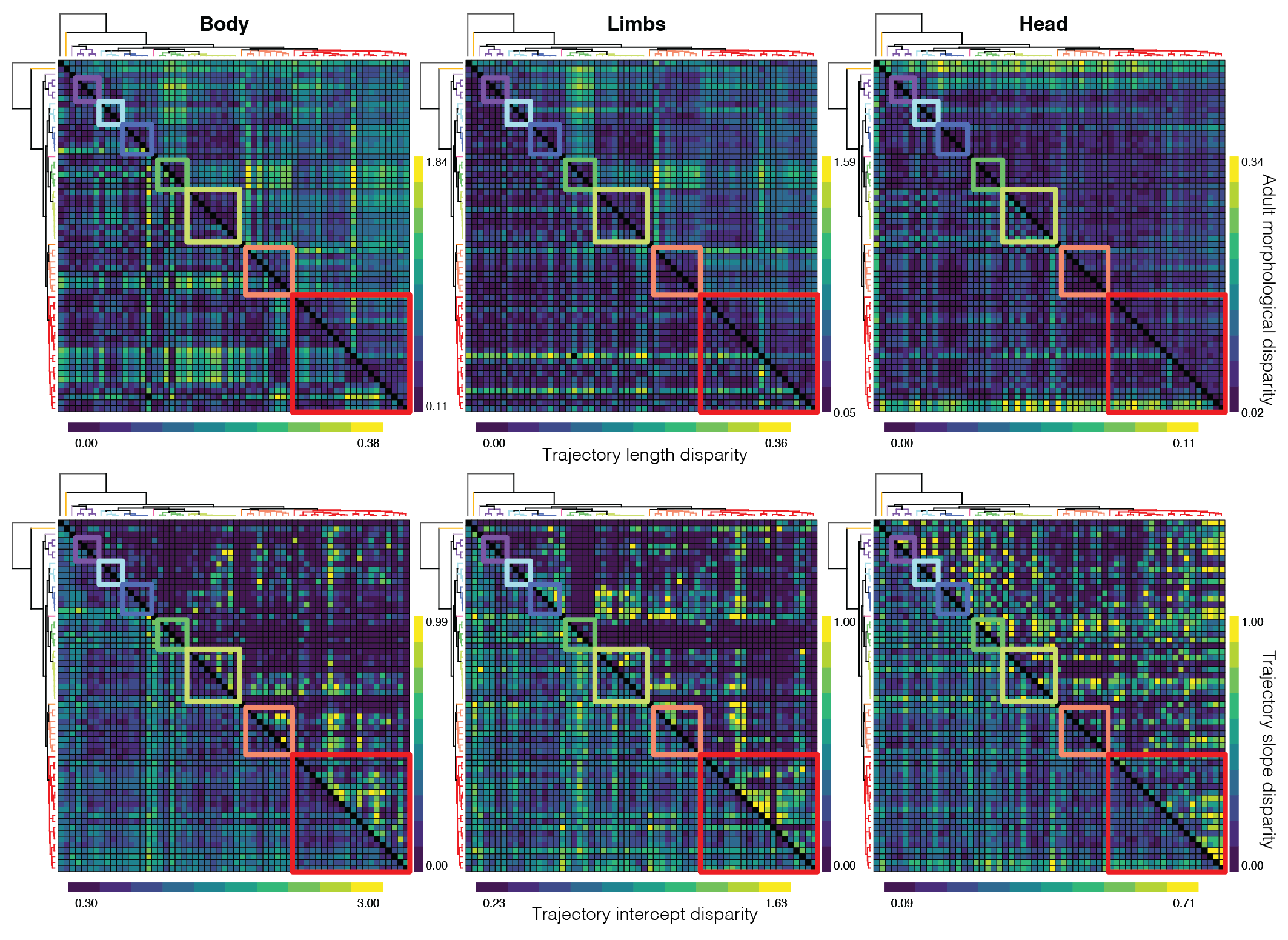


**Figure S9.** Morphological and ontogenetic disparity. Each grid is a square matrix where cells represent a pairwise comparison between species (diagonal in black). The phylogenetic tree depicting interspecific relationships is shown in the axes. Squares with colored borders indicate comparisons within clades (colors follow Fig. 3). For each species pair, we characterized morphological disparity as the Euclidean distance between the predicted adult phenotypes, disparity in trajectory lengths as the absolute difference between the estimated lengths, disparity in trajectory slopes as degrees, and disparity in trajectory intercepts as the Euclidean distance between intercepts.


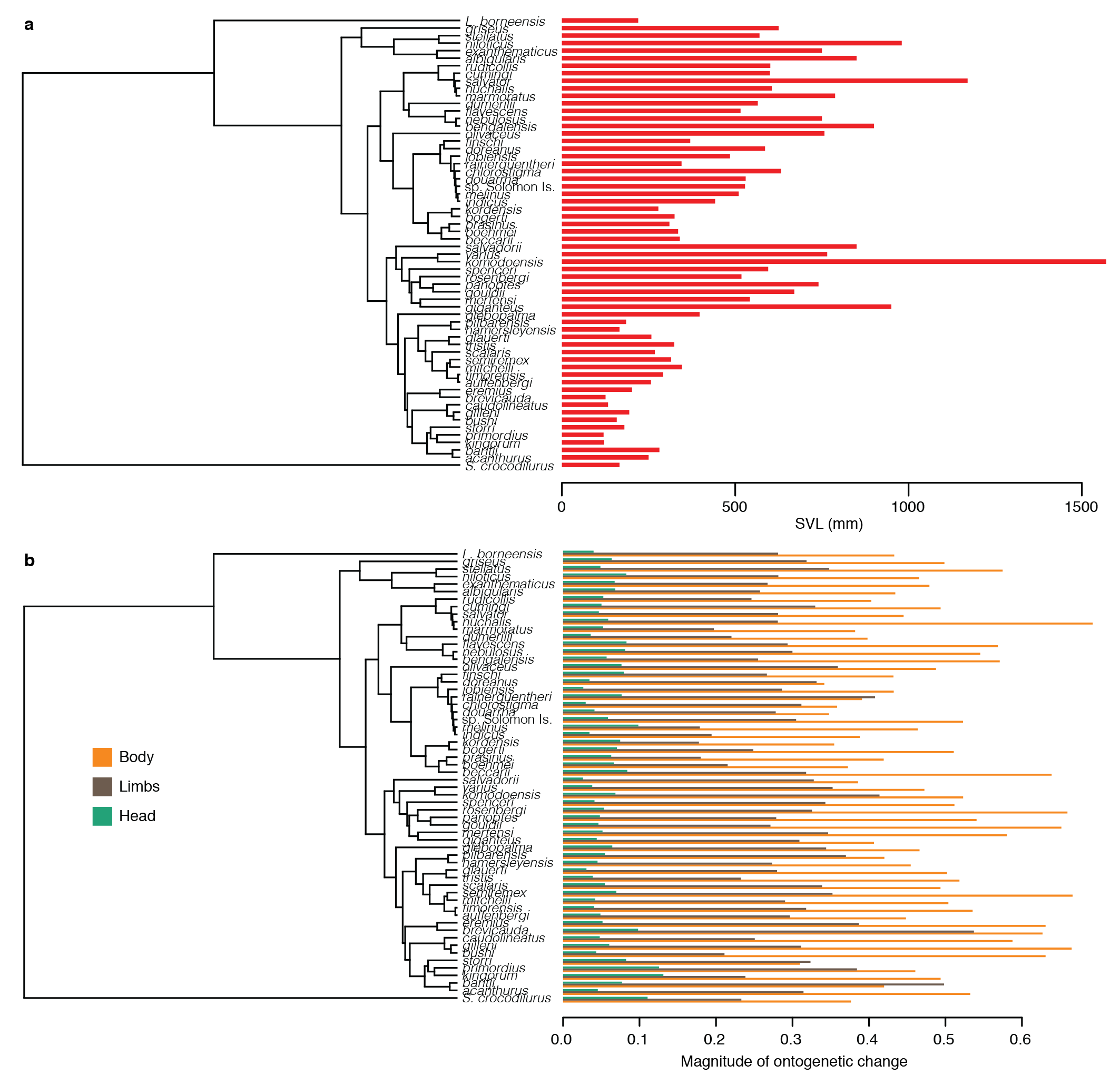


**Figure S10.** Relationship between body size and the magnitude of ontogenetic shape change. a) Maximum snout-vent length, a commonly used proxy for body size (SVL). b) Magnitude of ontogenetic shape change for each of the analyzed datasets.


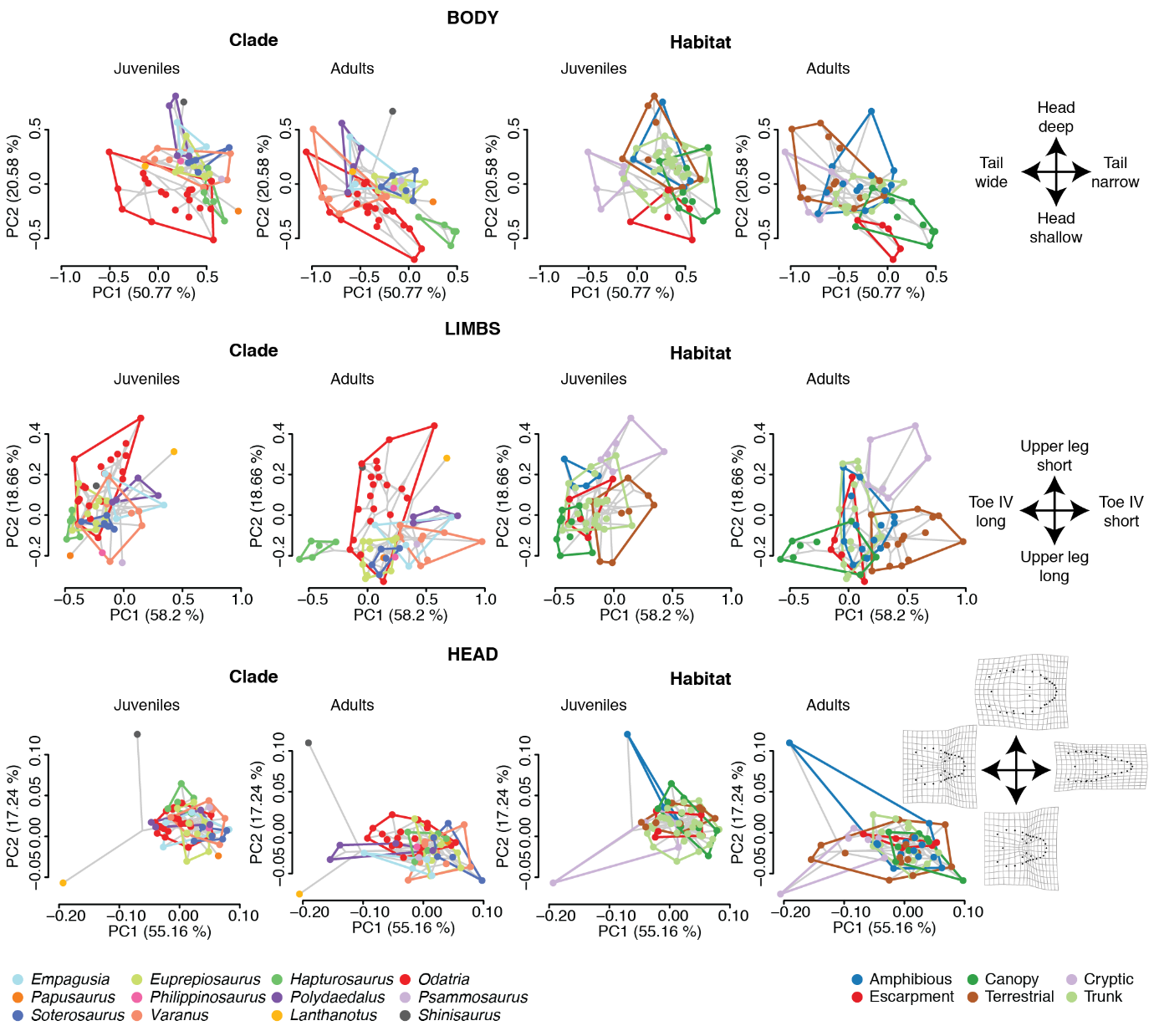


**Figure S11.** Phylomorphospace of juvenile and adult paleoanguimorphs. Axes represent the first and second principal components of the morphological data. Points (representing mean values per species) and convex hulls are colored by either clade or habitat use. For the linear measurements, the diagrams on the right indicate change in the traits that contribute the most to variation along each axis. For head shape, deformation grids show the head shape at the extremes of each axis and how they differ from the mean head shape.

**
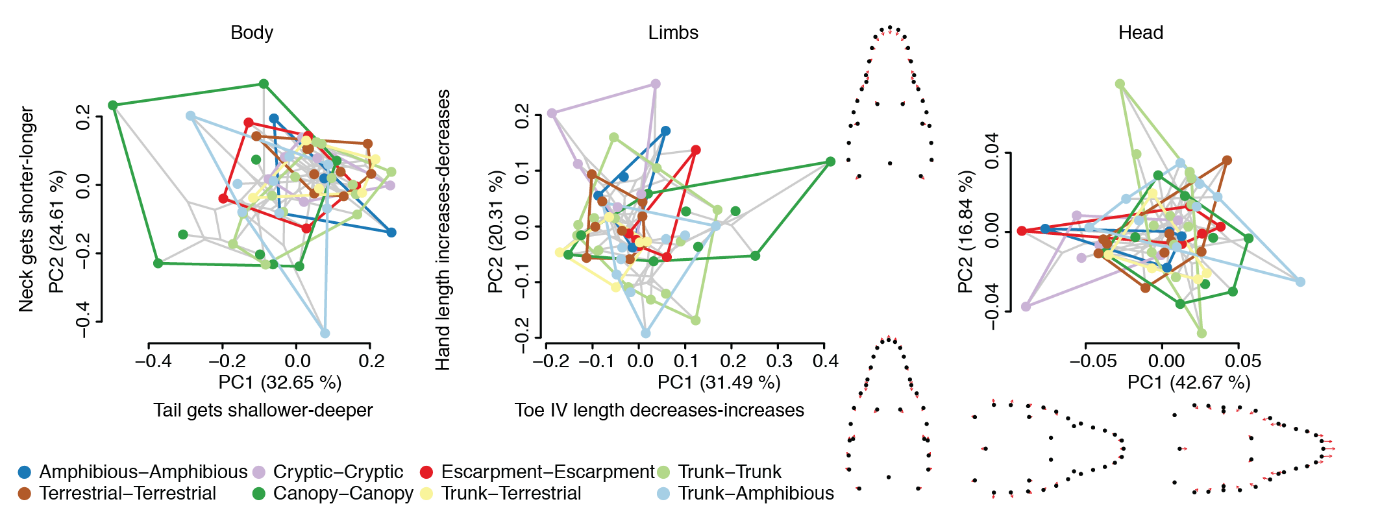
**

**Figure S12.** Phyloallomspace of Paleoanguimorpha. Axes correspond to the first two principal components (PCs) of the slopes of the ontogenetic allometric trajectories. The phylogenetic tree and inferred ancestral conditions (nodes) are shown in light gray. For the linear measurements, we show the trait whose slope contributes majorly to each PC and how it changes ontogenetically at the lower and upper extremes of each axis (separated by dash, in that order). For head shape, we show the average landmark configuration of juveniles of the species at each extreme (black points) and how landmarks move as each species grows (red arrows). Convex hulls are shown for each habitat use category.


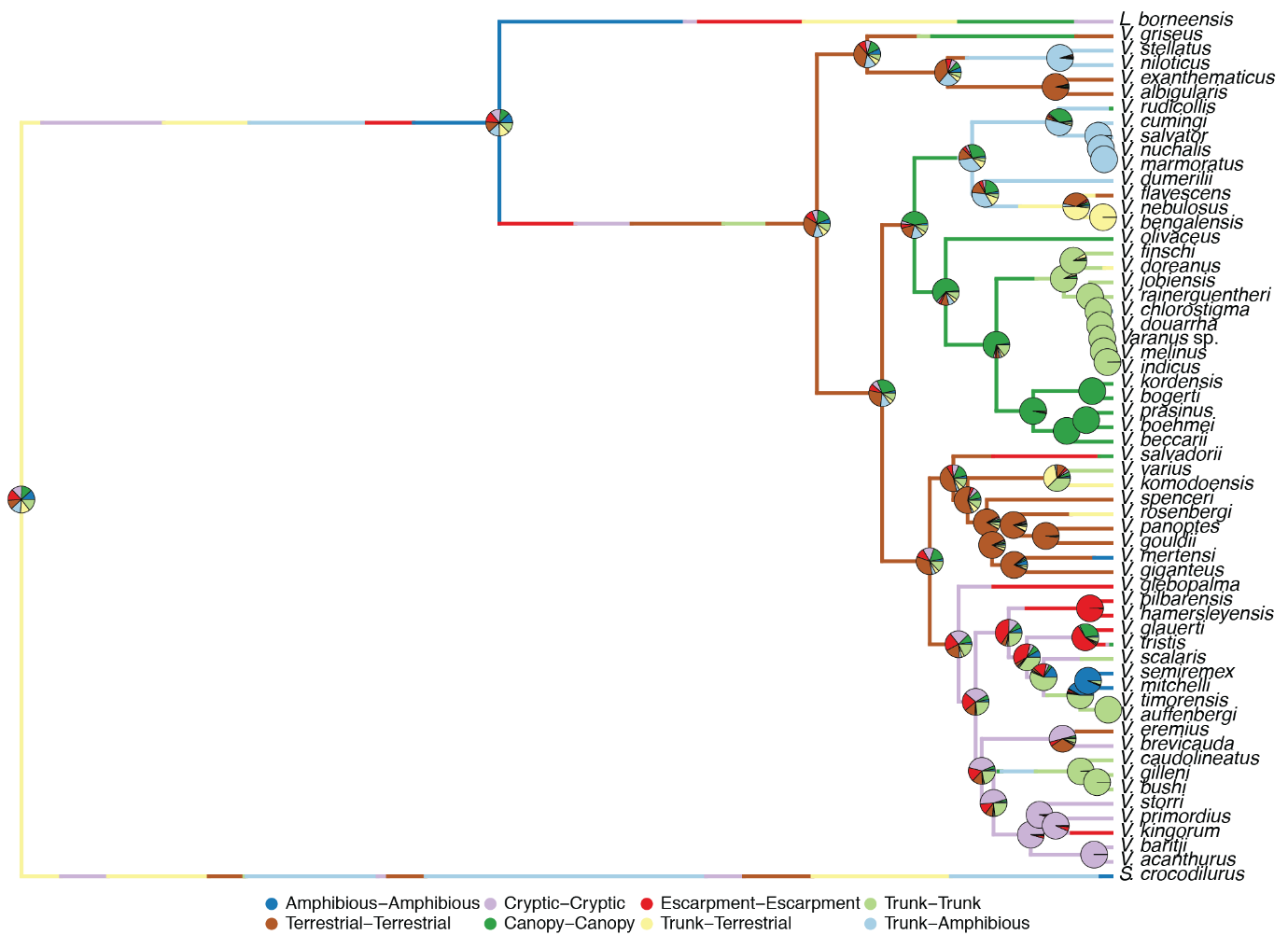


**Figure S13.** Stochastic character mapping of habitat use in Paleoanguimorpha. Branches are colored based on a single stochastic map used in downstream analyses. Pie charts indicate uncertainty in the reconstruction based on 1,000 stochastic maps.

**
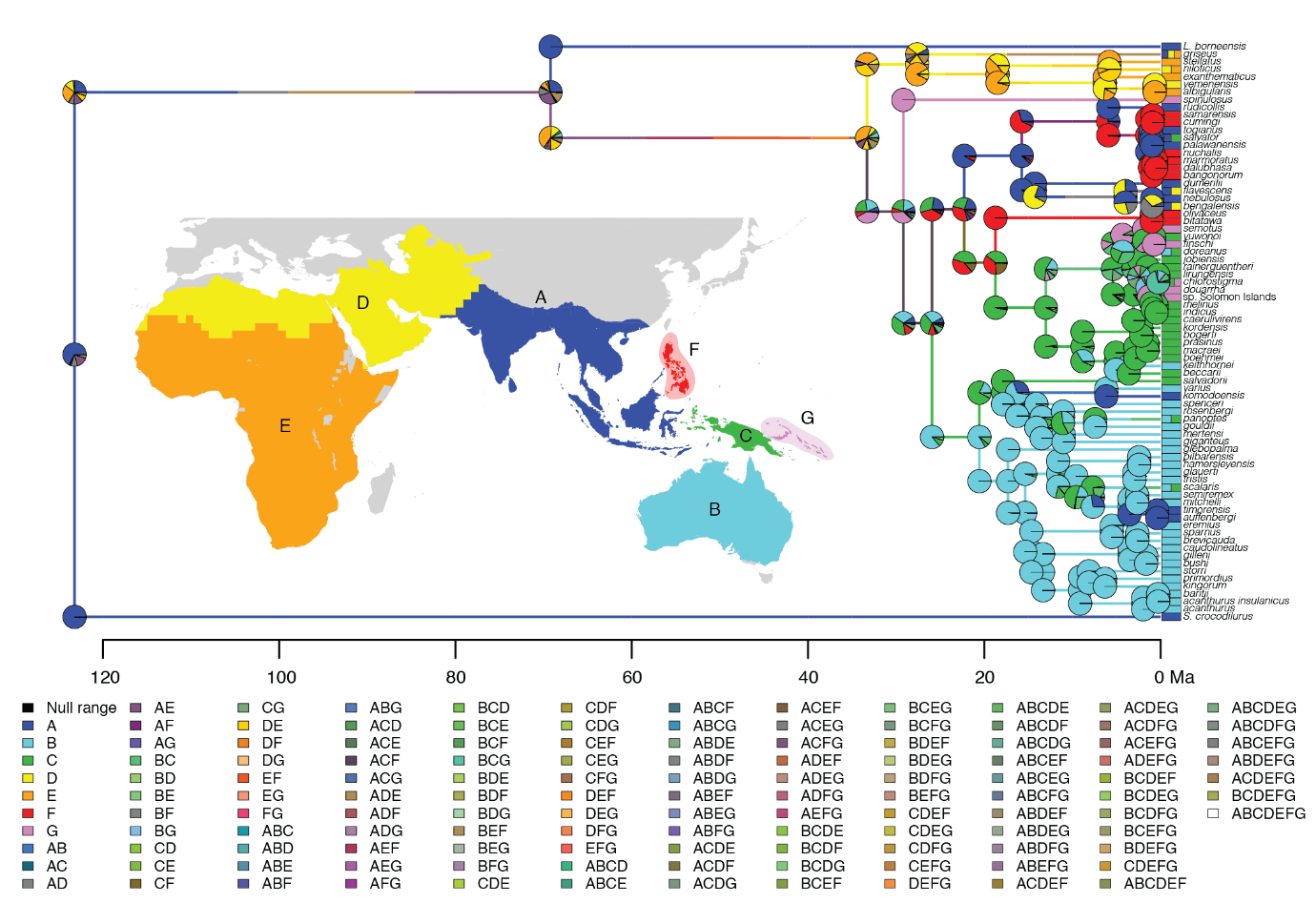
**

**Figure S14**. Ancestral range reconstruction of Paleoanguimorpha from Pavón-Vázquez et al. (in press). Branches are colored based on a single stochastic map used in downstream analyses.

**Supporting References**

*Includes references in Additional file 1.

Adams, D.C., and E. Otárola‐Castillo. 2013. geomorph: an R package for the collection and analysis of geometric morphometric shape data. Methods Ecol. Evol. 4:393–399.

Aplin, K. P., A. J. Fitch, and D. J. King. 2006. A new species of *Varanus* Merrem (Squamata: Varanidae) from the Pilbara region of Western Australia, with observations on sexual dimorphism in closely related species. Zootaxa 1313:1–38.

Böhme, W., H. J. Jacobs, T. Koppetsch, and A. Schmitz. 2019. The Kei Islands monitor lizard (Squamata: Varanidae: *Varanus*: *Euprepiosaurus*) as a distinct morphological, taxonomic, and conservation unit. Russ. J. Herpetol. 26:272–280.

Bouckaert, R., J. Heled, D. Kühnert, T. Vaughan, C. Wu., D. Xie, M. A. Suchard, A. Rambaut, and A. J. Drummond. 2014. BEAST 2: a software platform for Bayesian evolutionary analysis. PLoS Comput. Biol. 10:e1003537.

Brennan, I. G., A. R Lemmon, E. M. Lemmon, D. M. Portik, V. Weijola, L. Welton, S. C. Donnellan, and J. S. Keogh. 2020. Phylogenomics of monitor lizards and the role of competition in dictating body size disparity. Syst. Biol. 70:120–132.

Bucklitsch, Y., W. Boehme, and A. Koch. 2016. Scale morphology and micro-structure of monitor lizards (Squamata: Varanidae: *Varanus* spp.) and their allies: implications for systematics, ecology, and conservation. Zootaxa 4153:1–192.

del Canto, R. 2007. Notes on the occurrence of *Varanus auffenbergi* on Roti Island. Biawak 1:24–25.

Eidenmüller, B., A. Koch, J. Köhler, and R. Wicker. New findings on the relationships among New Guinea tree monitor lizards of the *Varanus prasinus* (Schlegel, 1839) complex. Hepetozoa 30:9–20.

Ewart, A. 1988. Geological history of the Fiji–Tonga–Samoan region of the SW Pacific, and some palaeogeographic and biogeographic implications. *In* J. P. Duffels (ed.). The cicadas of the Fiji, Samoa and Tonga Islands, their taxonomy and biogeography (Homeoptera, Cicadoidea). Scandinavian Science Press, Leiden, ZH.

Fitch, A. J., A. E. Goodman, and S. C. Donnellan. 2006. A molecular phylogeny of the Australian monitor lizards (Squamata: Varanidae) inferred from mitochondrial DNA sequences. Aust. J. Zool. 54:253–269.

Greer, A. E. 2001. Distribution of maximum snout‐vent length among species of scincid lizards. J. Herpetol. 35:383–395.

Gunz, P., P. mitteroecker, S. Neubauer, G. W. Weber, and F. L. Bookstein. 2009. Principles for the virtual reconstruction of hominin crania. J. Hum. Evol. 57:48–62.

Hall, R. 1996. Reconstructing Cenozoic SE Asia. Geol. Soc. Spec. Publ. 106:153–184.

Hall, R. 1998. The plate tectonics of Cenozoic SE Asia and the distribution of land and sea. *In* Hall, R., and J. D. Holloway. Biogeography and geological evolution of SE Asia. Backhuys Publishers, Leiden, ZH.

Holt, B. G., J. P. Lessard, M. K. Borregaard, S. A. Fritz, M. B. Araújo, D. Dimitrov, P-H. Fabre, C. H. Graham, G. R. Graves, K. A. Jønsson, D. Nogués-Bravo, Z. Wang, R. J. Whittaker, J. Fjeldså, and C. Rahbek. 2013. An update of Wallace’s zoogeographic regions of the world. Science 339:74–78.

Jacobs, H. J. 2003. A further new emerald tree monitor lizard of the *Varanus prasinus* species group from Waigeo, West Irian (Squamata: Sauria: Varanidae). Salamandra 39:65–74.

Kalyaanamoorthy, S., B. Q. Minh, T. K. F. Wong, A. von Haeseler, and L. S. Jermiin. 2017. ModelFinder: fast model selection for accurate phylogenetic estimates. Nat. Methods 14:587–589.

Landis, M. J., N. J. Matzke, B. R. Moore, and J. P. Huelsenbeck. 2013. Bayesian analysis of biogeography when the number of areas is large. Syst. Biol. 62:789–804.

Lemmon, A. R., S. A. Emme, and E. Moriarty Lemmon. 2012. Anchored hybrid enrichment for massively high-throughput phylogenomics. Syst. Biol. 61:727–744.

Lin, L., and J. J. Wiens. 2017. Comparing macroecological patterns across continents: evolution of climatic niche breadth in varanid lizards. Ecography 40:960–970.

Lucky, A., and E. M. Sarnat. 2010. Biogeography and diversification of the Pacific ant genus *Lordomyrma* Emery. J. Biogeogr. 37:624–634.

Maryan, B., P. M. Oliver, A. J. Fitch, and M. O’Connell. 2014. Molecular and morphological assessment of *Varanus pilbarensis* (Squamata: Varanidae), with a description of a new species from the southern Pilbara, Western Australia. Zootaxa 3768:139–158.

Matzke, N. J. 2013. Probabilistic historical biogeography: new models for founder-event speciation, imperfect detection, and fossils allow improved accuracy and model-testing. Front. Biogeogr. 5:242–248.

Matzke, N. J. 2014. Model selection in historical biogeography reveals that founder-event speciation is a crucial process in island clades. Syst. Biol. 63:951–970.

McCoy, M. 2006. Reptiles of the Solomon Islands. Pensoft Publishers, Sofia, SOF.

Meiri, S. 2018. Traits of lizards of the world: variation around a successful evolutionary design. Global Ecol. Biogeogr. 27:1168-1172.

Müller, R. D., J. Cannon, X. Qin, R. J. Watson, M. Gurnis, S. Williams, T. Pfaffelmoser, M. Seton, S. H. J. Russell, and S. Zahirovic. 2018. GPlates: building a virtual Earth through deep time. Geochem. Geophy. Geosy. 19:2243–2261.

Pavón-Vázquez, C. J., I. G. Brennan, A. Skeels, and J. S. Keogh. 2021. Competition and geography underlie speciation and morphological evolution in Indo-Australasian monitor lizards. Evolution (in press).

Pianka, E., and D. R. King (eds.). 2004. Varanoid lizards of the world. Indiana University Press, Bloomington, IN.

Ree, R. H., and S. A. Smith. 2008. Maximum likelihood inference of geographic range evolution by dispersal, local extinction, and cladogenesis. Syst. Biol. 57:4–14.

Revell, L. J. 2012. phytools: an R package for phylogenetic comparative biology (and other things). Methods Ecol. Evol. 3:217–223.

Ronquist, F. 1997. Dispersal-vicariance analysis: a new approach to the quantification of historical biogeography. Syst. Biol. 46:195–203.

Sabaj M.H. 2019. Standard symbolic codes for institutional resource collections in herpetology and ichthyology: an online reference. Version 7.1 (21 March 2019). Available at http://www.asih.org/. Accessed December 1, 2020.

Sherratt, E., A. R. Rasmussen, and K. L. Sanders. 2018. Trophic specialization drives morphological evolution in sea snakes. Roy. Soc. Open Sci. 5:172141.

Smith, L. A., S. S. Sweet, and D. R. King. 2004. *Varanus scalaris*. *In* Pianka, E. R., and D. R. King (eds.). Varanoid Lizards of the World. Indiana University Press, Bloomington, IN.

Stamps, J. A., and R. M. Andrews. 1992. Estimating asymptotic size using the largest individuals per sample. Oecologia, 92:503–512.

Stekhoven, D.J., and P. Bühlmann. 2012. MissForest—non-parametric missing value imputation for mixed-type data. Bioinformatics 28:112–118.

van Schingen, M., C. T. Pham, H. A. Thi, T. Q. Nguyen, M. Bernardes, M. Bonkowski, and T. Ziegler. 2015. First ecological assessment of the endangered crocodile lizard, *Shinisaurus crocodilurus*, Ahl, 1930 in Vietnam: microhabitat characterization and habitat selection. Herpetol. Conserv. Biol. 10:948–958.

Weijola, V. S-Å. 2010. Geographical distribution and habitat use of monitor lizards of the north Moluccas. Biawak 4:7–23.

Weijola, V., and S. S. Sweet. 2015. A single species of mangrove monitor (*Varanus*) occupies Ambon, Seram, Buru and Saparua, Moluccas, Indonesia. Amphib. Reptile Conserv. 9:14–23.

Weijola, V., F. Kraus, V. Vahtera, C. Lindqvist, and S. C. Donnellan. 2017. Reinstatement of *Varanus douarrha* Lesson, 1830 as a valid species with comments on the zoogeography of monitor lizards (Squamata: Varanidae) in the Bismarck Archipelago, Papua New Guinea. Austr. J. Zool. 64:434-451.

Weijola, V., V. Vahtera, A. Koch, A. Schmitz, and F. Kraus. 2020. Taxonomy of Micronesian monitors (Reptilia: Squamata: *Varanus*): endemic status of new species argues for caution in pursuing eradication plans. Roy. Soc. Open Sci. 7:200092.

Weijola, V., V. Vahtera, C. Lindqvist, and F. Kraus. 2019. A molecular phylogeny for the Pacific monitor lizards (*Varanus* subgenus *Euprepiosaurus*) reveals a recent and rapid radiation with high levels of cryptic diversity. Zool. J. Linn. Soc. 186:1053–1066.
